# An empirically informed agent-based model of a Nepalese smallholder village

**DOI:** 10.1016/j.mex.2021.101276

**Published:** 2021-03-31

**Authors:** Nicholas Roxburgh, Andrew Evans, Raj K. GC, Nick Malleson, Alison Heppenstall, Lindsay Stringer

**Affiliations:** a School of Earth and Environment, University of Leeds, Leeds LS2 9JT, UK; b School of Geography, University of Leeds, Leeds LS2 9JT, UK; c School of Public and International Affairs, Virginia Polytechnic Institute and State University, Blacksburg, VA 24060, USA; d Department of Environment & Geography, University of York, Heslington, York YO10 5NG, UK

**Keywords:** Simulation, Agriculture, Livestock, Socio-ecological system, Stressors, Shocks, Earthquakes, Multi-scale, Population synthesis, Nepal

## Abstract

Agent-based modelling methodologies offer a number of advantages when it comes to socio-ecological systems research. In particular, they enable experiments to be conducted that are not practical or feasible to conduct in real world settings; they can capture heterogeneity in agent circumstances, knowledge, behaviour, and experiences; and they facilitate a multi-scale, causal understanding of system dynamics. However, developing detailed, empirically informed agent-based models is typically a time and resource intensive activity. Here, we describe a detail-rich, ethnographically informed agent-based model of a Nepalese smallholder village that was created for the purpose of studying the impact of multiple stressors on mountain communities. In doing so, we aim to make the model accessible to other researchers interested in simulating such communities and to provide inspiration for other socio-ecological system modellers.

• The model is described using the ODD protocol.
• The number of replicate runs required for experiments is discussed, and the model validation and sensitivity analysis processes that have been conducted are explained.
• Suggestions are made for how the model can practically be used and for how model outputs can be analysed.

## Graphical abstract

**Figure:**
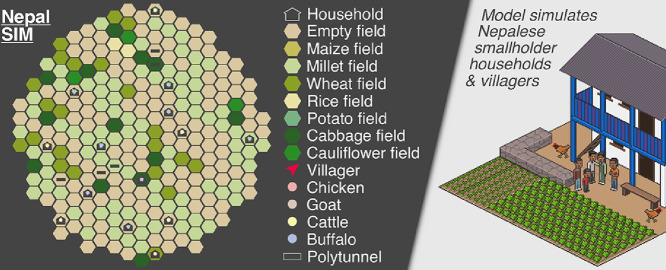

Specifications table

**Table:** 

Subject Area:	Environmental Science
More specific subject area:	*Social simulation*
Method name:	*Nepal Stressor Interaction Model (Nepal SIM)*
Name and reference of original method:	*NA*
Resource availability:	*NetLogo is available from:*https://ccl.northwestern.edu/netlogo/*R is available from:*https://www.r-project.org

*Method details

## 1. Method details

In this paper, we describe an empirically informed agent-based model (ABM) of a rural Nepalese village. The original purpose of the model was to simulate the impact of multiple stressors on mountain people and households [24,26]. However, it can potentially be adapted for the study of other topics or be used as inspiration in the design of other models. The version set out here places particular emphasis on the impact of extreme weather events, earthquakes, and falling fertility rates on household finances and village demographics, though other stressor could be readily incorporated. Furthermore, with minor adaptations, the model could be used to study additional stressor impact domains. For example, subject to some minor changes, the impact of stressors on food security dynamics could be examined. The model could also be adapted to simulate other smallholder communities affected by multiple stressors. That said, the model includes a substantial number of parameters that would need to be updated, and some of the model processes may also need reconfiguring to account for case study particularities. Particular strengths of the ABM include: (a) the fact that it runs at daily timesteps, enabling socio-ecological dynamics to be studied at high temporal resolution; (b) the multiple scales at which processes can be observed – individual, household, and village; (c) its empirically informed nature; and (d) its unusually detailed depiction of demographic processes, livelihoods, and villager finances.

Here, we describe the ABM using the ODD protocol [7,8]. This is a standardised protocol for describing ABMs, consisting of three main parts: An Overview section; a Design concepts section; and a Details section. The design decisions and parameters that are documented in it are primarily informed by fieldwork conducted by the authors in a village in Dolakha, Nepal, in 2015 and 2017 – a village that is populated by members of the Tamang ethnic group. The fieldwork methods that were used are detailed in Roxburgh [24]. They include household surveys, the participatory development of a village wiki, and focus groups. The latter comprised sessions discussing community history, institutions, seasonal calendars, resource flows, decision-making, typical life courses, young villagers’ experiences and expectations, and multiple stressors. Other sources are occasionally drawn upon as well, and certain parameters have undergone calibration. Where the latter two cases apply, the source or rationale is stated.

In addition to the ODD protocol, the paper discusses the number of replicates necessary when conducting experiments using the model (see *Section 3*), it details the model validation (see Section 4) and sensitivity analysis (see *Section 5*) that was conducted for the original study, and it provides practical guidance for using the model (see *Section 6*), adapting the model (see *Section 7*), and analysing the outputs (see *Section 8*). Additional discussion of the model can be found in Roxburgh [24]. In this paper, the study site is referred to by the pseudonym, Namsa. Ethical approval for the study was granted by the University of Leeds (ref. AREA 14-103).

Relative to most socio-ecological ABM studies, the model can be considered on the descriptive side. It includes all of the main processes identified during fieldwork that influence village economics and demographics and that interact in a significant way with the three stressors that were considered in the original study that the model was designed for. These include: the 2015 Nepal Earthquake; changing fertility rates; and increasing crop variability. This means that there are a great many factors at play. However, the individual processes are depicted in a relatively parsimonious fashion. The aim is to capture important characteristics and behaviours of the system while avoiding creating something of Daedalian complexity.

**Fig. 1:**
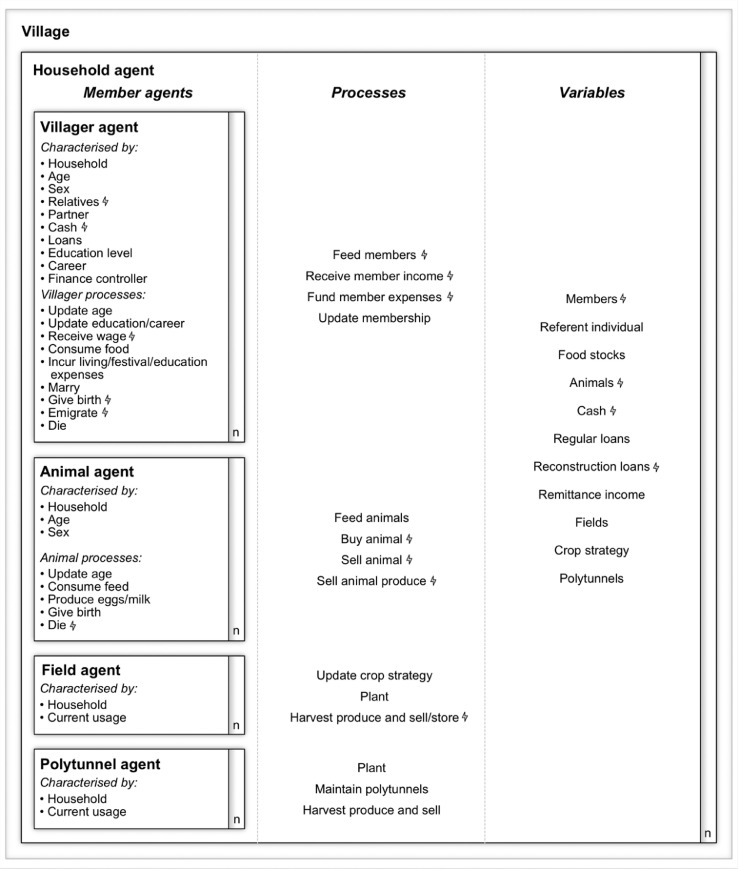
A conceptual diagram showing a simplified representation of agent variables, processes, and interactions. The lightning bolts show the variables and processes that are directly affected by the stressor scenarios presented in Sc [26].

A conceptual diagram showing a simplified representation of agent variables, processes, and interactions is provided in Fig. 1.

## 2. ODD Protocol

An outline of the ODD is shown in Table 1. Each sub-section has an associated code to aid cross-referencing.

**Table 1:** Outline of the adapted ODD protocol.

Sections	Sub-Sections	Section code
**Overview**	Model purpose	M
	Entities, state variables, and scales	E
	Process overview and scheduling	P
**Design concepts**	Theoretical and empirical background	C1
	Individual decision-making	C2
	Learning	C3
	Individual sensing	C4
	Individual prediction	C5
	Interaction	C6
	Collectives	C7
	Heterogeneity	C8
	Stochasticity	C9
	Observation	C10
**Details**	Sub-models	S
	Initialisation	I
	Scenarios	Sc

**M.** M**odel purpose**

The model was designed to simulate how multiple stressors could affect agrarian communities in the Mid-Hills of Nepal between 2015 and 2030. However, it could also be used to examine topics such as demographic and livelihood change, food security, and household economics in mountain communities. The model incorporates all of the main social and agricultural systems that were identified in Namsa and is populated with artificially intelligent agents designed to behave in ways that, as closely as possible, match how the villagers at the fieldsite behaved. Various stressor scenarios can be simulated within the model with each one combining a different set of assumptions about how stressors will play out. In the original iteration of the model, we focused on assessing the potential long-term impact of the 2015 earthquakes, changing fertility rates, and increasing crop yield variability on household finances and village demographics. These scenarios are detailed in section *Sc*.

## E. Entities, state variables, and scales

***E1*. Entities.** The model consists of eight kinds of entity: villagers, households, chickens, goats, cattle, buffalo, fields, and polytunnels.

• ***E1.1 Villagers*** are characterised by their sex, age, and household, their relatives, their personal cash and loans, their education level, their career, and their finance controller. The latter is an important concept within the model. It refers to the party within the villager's household who receives their income and funds their expenditure. Villagers who are a household referent (see E1.2), a wife of a referent, a parent(-in-law) or grandparent(-in-law) of a referent, a daughter or widowed daughter-in-law of a referent, or a still in education son of a referent, will have the household itself as their finance controller. In contrast, sons of referents who have completed their education manage their own income and expenditure, along with that of their wife and their children. This approximates how finances are managed in Namsa. Finally, villagers have variables stating the timing of their marriage and the timing of their death which are either determined at model initialisation (see I4 and I6) or at birth (see S5), and married women have additional variables stating their desired number of children and the timing of their next child's birth (see S5).
• ***E1.2 Households*** are characterised by their members, fields (some of which may be specialist paddy fields), polytunnels, livestock, cash, loans, a crop strategy, a referent individual, and potentially a monthly remittance income (see I11). The crop strategy is a list of the number of fields the household is provisionally allocating to each of the available crop types during the year ahead. The referent individual is the youngest adult (defined as being over-18 years of age) male who does not have siblings within the household. Alternatively, in the absence of an adult male without siblings, the referent is the youngest widowed adult female.^1^ The referent is a role invented purely to aid management of the demographic processes in the model. It is not an actual role in the village.
• ***E1.3 Chickens*** are characterised by the household they belong to, their age, their sex, and the stage they are at in their egg laying cycle (see P2) in the case of females.
• ***E1.4 Goats, cattle and buffalo*** are characterised by the household they belong to, their age and their sex.
• ***E1.5 Fields and polytunnels*** are characterised by the household they belong to and their current usage.

**E2. Globals.** The model includes a number of global variables which are accessible by all agents and processes in the model. These include past and present crop yields (see *I12*), forecast crop yields (see *S6*), produce prices and expense parameters (see Table 2), and scenario settings (see *Sc*). Details of how the parameters were determined are provided in Roxburgh [24].

**Table 2:** Model parameters and the methods used to determine them [24].

Parameter group	Parameter	Unit	Standard value	Method
Animal purchase price	Chicken purchase price	NPR	400	Wiki
	Goat purchase price	NPR	5000	Wiki
	Cattle purchase price	NPR	3000	Wiki
	Buffalo purchase price	NPR	10,000	Wiki
Animal slaughter price	Chicken slaughter price	NPR	1200	Wiki
	Goat slaughter price	NPR	7600	Wiki
	Female buff slaughter price	NPR	28,000	Wiki
	Male buff slaughter price	NPR	42,000	Wiki
Career advancement probability	Prob. of doing +2 (i.e. college) post-school	-	0.5	Young persons’ focus group
	Prob. of obtaining salaried job if completed +2 (i.e. college)	-	0.25	Young persons’ focus group
	Prob. of advancing to level II from level I salaried job	-	0.75	Typical life & young persons’ focus groups
	Prob. of advancing to level III from level II salaried job	-	0.5	Typical life & young persons’ focus groups
	Prob. of advancing to level IV from level III salaried job	-	0.5	Typical life & young persons’ focus groups
Subsistence crop yield	Standard maize yield	kg/ropani	84.8	Resource flows focus group
	Standard millet yield	kg/ropani	104.9	Resource flows focus group
	Standard wheat yield	kg/ropani	55.3	Resource flows focus group
	Standard rice yield	kg/ropani	150.1	Resource flows focus group
Cash crop yield	Standard potato yield	kg/ropani	793.1	Resource flows focus group
	Standard cabbage yield	kg/ropani	1,017.4	Resource flows focus group
	Standard cauliflower yield	kg/ropani	610.4	Resource flows focus group
Subsistence crop price	Maize price	NPR/kg	25	Wiki & resource flows focus group
	Millet price	NPR/kg	19	Wiki & resource flows focus group
	Wheat price	NPR/kg	22	Wiki & resource flows focus group
	Rice price	NPR/kg	20	Wiki & resource flows focus group
Cash crop price	Potato price	NPR/kg	22	Wiki & resource flows focus group
	Cabbage price	NPR/kg	18	Wiki & resource flows focus group
	Cauliflower price	NPR/kg	25	Wiki & resource flows focus group
Cottage industry income	Egg sale price	NPR/egg	7	Resource flows focus group
	Milk income from cow for one-person household	NPR/day	135	Wiki & survey
	Milk income from cow for two-person household	NPR/day	90	Wiki & survey
	Milk income from buff for one-person household	NPR/day	225	Wiki & survey
	Milk income from buff for two-person household	NPR/day	150	Wiki & survey
	Tomato tunnel income	NPR/harvest	4,140	Survey & resource flows focus group
Day-to-day expenses	Other living expenses	NPR	27	Survey
	Other food expenses	NPR	52	Survey & resource flows focus group
Educational expenses	Monthly school expenses	NPR	400	Wiki
	Monthly +2 expenses (i.e. college expenses)	NPR	800	Young persons’ focus group & wiki
Festival, funeral & wedding costs	Festival expenses	NPR	200	Survey & seasonal calendar focus group
	Wedding gift	NPR	100,000	Wiki
	Funeral cost	NPR	200,000	Wiki
Salaried job abroad	Foreign job salary	NPR/month	10,000	Young persons’ focus group & wiki
Salaried job in Nepal	Level I job salary	NRP/month	12,000	Typical life & young persons’ focus groups
	Level II job salary	NPR/month	15,000	Typical life & young persons’ focus groups
	Level III job salary	NPR/month	20,000	Typical life & young persons’ focus groups
	Level IV job salary	NPR/month	25,000	Typical life & young persons’ focus groups
Short-term labouring	Short-term labouring wage	NPR/day	500	Wiki
	Daily short-term labouring probability	-	0.19	Survey & seasonal calendar focus group
Remittance	Remittance income	NPR/month	10,000	Survey

**E3. Scales.** Each field equates to half a ropani of land (254.35 m^2^), each polytunnel equates to 68.34 m^2^, and one time-step corresponds to one day.^2^ Simulations run for 5,475 time-steps which equates to fifteen years. For simplicity, leap days are not accounted for in the model. The simulations begin on 1 January 2015 and end on 31 December 2029.

**Fig. 2:**
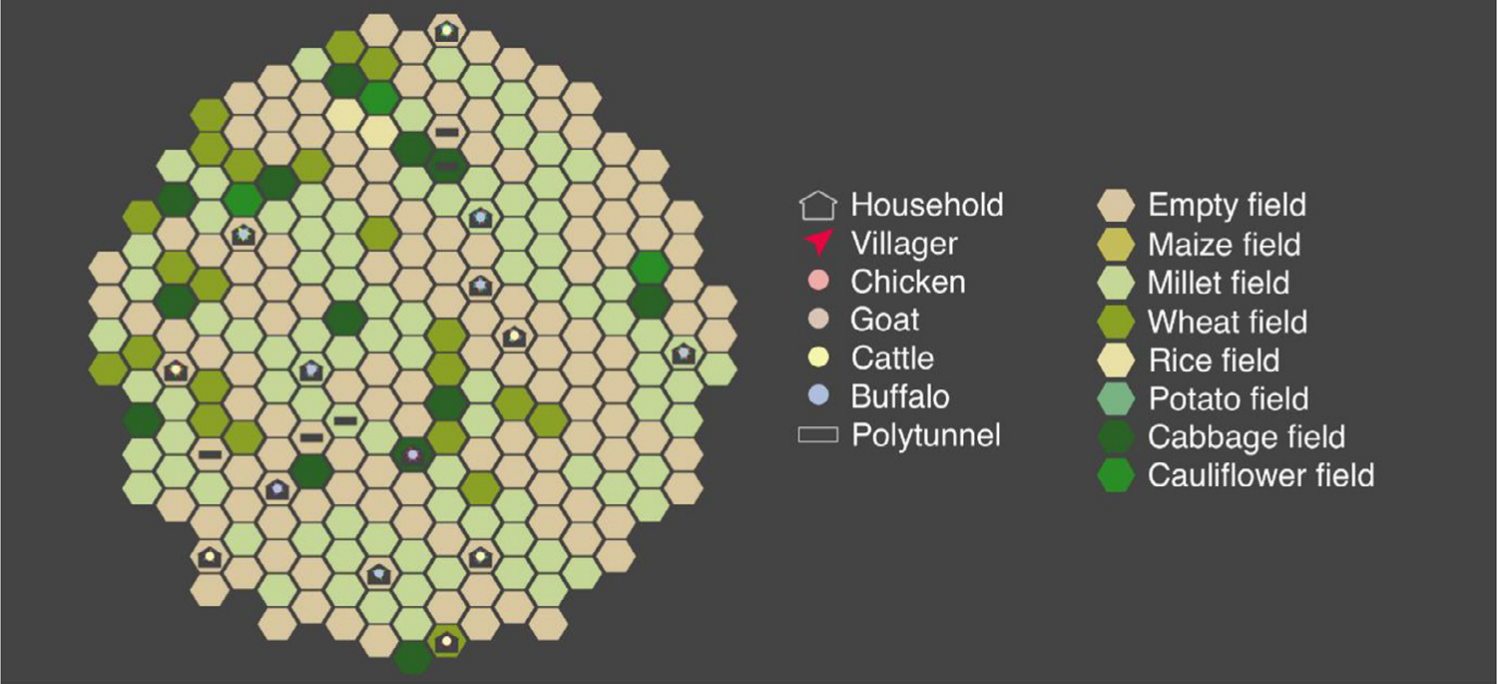
An example snapshot of the model during October in the first year of one of the runs, as visualised in NetLogo.

**E4. Visualisation.** For visualisation purposes, fields are represented by hexagons, households by house symbols, villagers by arrowheads, animals by circles that are coloured according to species, and polytunnels by grey rectangles (see Fig. 2). Although the relative location of the agents is meaningless in respect to model dynamics, fields are clustered by the household they belong to in order to aid visual interpretation and households, villagers, and animals are placed together on a randomly chosen field that is owned by their household. Polytunnels are placed on a separately chosen household field.

## P. Process overview and scheduling

**P1. Villagers.** On each time-step, villagers age by one day. A check is then performed on each villager to see whether they have reached an education or career juncture. If they have, their education status and/or career is updated (see *S1*). Next, a check is performed to determine whether villagers are due to receive a salary, a wage, or a pension on the current time-step. Villagers with salaried jobs are paid at the end of each month at the rate shown in Table 3, while villagers who are engaged in short-term labouring are paid NPR 500 on the days that they work. Whether or not short-term labourers work on a given day is determined stochastically. By default, work opportunities are assumed to be available on 19% of non-festival days^3^ and are never declined. Villagers who are entitled to a pension, meanwhile, will receive NPR 10,000 on the first day of each month. Any income received will be transferred to the villager's finance controller (see *E1.1*).

**Table 3:** Monthly salary by job type.

Job type	Monthly salary (NPR)
Salaried job abroad	10,000
Salaried job in Nepal (Level 1)	12,000
Salaried job in Nepal (Level 2)	15,000
Salaried job in Nepal (Level 3)	20,000
Salaried job in Nepal (Level 4)	25,000

Next the personal expenditure of each villager – which is paid for by their finance controller – is determined. Each day, villagers who are not abroad will incur food expenses and non-specific other living expenses^4^ – the latter is fixed at NPR 27 by default, while the former varies depending on personal circumstances (see *S2*). On the first day of each month, villagers who are attending school also incur an expense of NPR 400, while those who are attending college incur an expense of NPR 800. Should the day be one of the 23 festival days^5^ that are celebrated in Namsa, each villager who is not working abroad will incur an additional expense of NPR 320. Next, villagers who are their own finance controller will assess their monetary situation (see *S11*). As part of this assessment, the villager will determine how many portions of meat he and his dependents can afford to consume each week. After these financial matters have been processed, checks are performed for each villager to see whether they are due to die (see *S3*), to marry (see *S4*), or to give birth (see *S5*) on the current time-step.

**P2. Chickens.** On each time-step, chickens age by one day and consume 32g of maize, 32g of millet and 10g of wheat, the cost of which is deducted from the cash stock of their household. When a female chicken reaches six months of age, it will begin a cycle of laying an egg a day for thirty days, followed by three months of no eggs. These eggs are each sold for NPR 7 by the chicken's household. This money is added to the household's cash stock. Male chickens will be slaughtered when six months old (183 days), while female chickens will be slaughtered when five years old (1,825 days old). It is assumed that the carcass is sold for NPR 1,500 and a replacement 14-day-old chick is purchased for NPR 400. The net income is added to the household's cash stock. In the real world, there is of course more variability in poultry life courses and economics. The processes and parameters set out here are designed to represent what is typical. This is also true for the livestock.

**P3. Goats.** On each time-step, goats age by one day and consume 25g of maize and 25g of millet,^6^ the cost of which is deducted from the cash stock of their household. At 329 days of age and 612 days of age, females will give birth – 62.2% of the births will yield one kid, 33.3% will yield two kids, and 4.4% will yield three kids. It is assumed that the kids are sold immediately for NPR 5,000 each. The adult goats are sold at two years of age for NPR 8,800 and a replacement 112-day-old goat is purchased for NPR 5,000. The net income is added to the household's cash stock.

**P4. Female Cattle (Cows).** On each time-step, cattle age by one day and consume 240g of maize and 240g of millet, the cost of which is deducted from the cash stock of their household. At 913, 1461, 2009, 2557, 3105, 3653, and 4201 days of age, cows will give birth to one calf. The calves are sold for NPR 3,000. Cows will provide milk each day post-pregnancy up until two months before they next give birth or until they reach 4,688 days of age. It is implicitly assumed that some of this milk is consumed within the household, but households with just one or two members are assumed to have surplus to sell. One-member households receive NPR 135 per day for selling their surplus milk, while households with two members receive NPR 90. This money is added to the household's cash stock. Cows will die upon reaching 18 years of age and are replaced by a 548-day-old calf at a cost of NPR 3,000.

**P5. Male Cattle (Oxen).** On each time-step, cattle age by one day and consume 240g of maize and 240g of millet, the cost of which is deducted from the cash stock of their household. Oxen will die upon reaching 18 years of age and are replaced by a 548-day-old calf at a cost of NPR 3,000.

**P6. Buffalo.** On each time-step, buffalo age by one day and consume 280g of maize and 280g of millet, the cost of which is deducted from the cash stock of their household. At 1642, 2220, 2798 and 3376 days of age, female buffalo will give birth. The calves will be sold immediately for NPR 10,000. This money is added to the household's cash stock. Female buffalo will provide milk each day post-pregnancy up until three months before they next give birth or are slaughtered. As with the cows, it is implicitly assumed that some of this milk is consumed within the household, but households with just one or two members are assumed to have surplus to sell. One-member households receive NPR 225 per day for selling their surplus milk, while households with two members receive NPR 150. This money is added to the household's cash stock. Female buffalo will be slaughtered at 10 years of age, and males will be slaughtered at 14 years of age. Their meat will be sold for NPR 26,500 in the case of males, and NPR 19,875 in the case of females. They are immediately replaced by a 548-day-old calf at the cost of NPR 10,000. The net income is added to the household's cash stock.

**P7. Households.** Households begin each time-step by checking whether the current day is a crop plantation or harvesting day (see Table 4). If it is the former, the household will re-evaluate its existing crop strategy in order to determine how many fields, if any, it wishes to allocate to the relevant crop (see *S6*). Once this is known, the particular fields that are to be allocated to the crop are selected randomly from those that are not currently in use, except in the case of rice cultivation which is only done in the specialist paddy fields. Plantation then takes place (see *S7*). If the current day is instead a harvesting day, fields will be harvested, and crops will be sold (see *S8*). The newly harvested fields will then become available for planting once again.

**Table 4:** Crops that are included in the model and their associated details. These values were determined during the fieldwork resource flows focus group and by the wiki authors during the fieldwork that informed the model [24]. They represent agreed averages.

Crop	Timing of plantation (day of the year)	Seed requirement (kg per ropani or NPR per ropani)	Cost of fertiliser / pesticide (NPR per ropani)	Timing of harvest (day of the year)	Standard yield (kg per ropani)	Market price (NPR per kg)
Potato	29^th^	110 kg	627	170^th^	793.1	22
Maize	57^th^	0.8 kg	313	256^th^	84.8	25
Millet	166^th^	1.2 kg	313	334^th^	104.9	19
Wheat	275^th^	6.4 kg	313	127^th^	55.3	22
Rice	174^th^	28.8 kg	313	342^nd^	150.1	20
Cabbage	219^th^	300 NPR	1506	15^th^	1017.4	18
Cauliflower	244^th^	300 NPR	764	19^th^	610.4	25

After crops have been dealt with, the next step for households is to check whether their referent needs to be updated. The conditions set out earlier for selecting the referent determine whether this is the case (see *E1.2*). Following this, the households check whether the finance controller of each of their members needs updating. Again, the conditions set out earlier for determining the finance controller of villagers determine whether this is the case (see *E1.1*). After this, should the time-step correspond to the first day of a month, households that are designated as being in receipt of a remittance (see *I11*) will receive the said remittance. This is worth NPR 10,000 per month. Next, households check whether any of their members have met the conditions necessary to trigger household fission and then whether their circumstances have changed such that they need to buy or sell livestock and/or poultry (see *S9* and *S10*). Finally, households assess the state of their finances in light of the income and/or outgoings that have taken place earlier in the time-step (see *S11*). As part of this last process, the household will determine how many portions of meat that those with their household as their finance controller can afford to eat each week. Meat is modelled as it represents the main luxury expenditure outside of festival times.

**P8. Polytunnels.** Polytunnels are used to grow tomatoes. In the model, they undergo maintenance on the 46^th^ day of the year. When this happens, households incur a cost of NPR 700. Planting then occurs on the 98^th^ day of the year. Seeds cost NPR 100 per tunnel, while pesticide and fertiliser collectively cost NPR 770 per tunnel. Harvesting occurs fortnightly from the 213^th^ day of the year until 44^th^ day of the next year. Each fortnightly harvest is worth NPR 4,140 per tunnel.

## C. Design Concepts

**C1. Theoretical and empirical background.** The design and parameterisation of the model has primarily been informed by empirical evidence gathered during fieldwork in Namsa [24]. However, in cases where it was not possible to parameterise the model using first-hand data alone, third party datasets have also been drawn upon. For example, the demographic sub-models make use of marriage data from the Nepal Demographic and Health Survey [16], fertility data from Karkee and Lee [10], and mortality data from the WHO [30] life tables for Nepal.

**C2. Individual decision-making.** When it comes to depicting specific decision domains, such as crop and livestock choice or the management of household finances, the model in many cases seeks to mimic in a simplified, caricatured fashion, the real-world decision-making process described by the villagers at the fieldsite. Often these decision-processes are based on well-established heuristics or customs, but occasionally they involve agents making more complex calculations. For example, crop mix, loan repayment, and meat consumption decisions involve the agents taking into account forecasts about how the future may play out. In a few instances, real world decisions are instead modelled in a simple probabilistic fashion. The change points in the village life course are examples of this.

The model assumes that people tend towards what we were told represents typical behaviour in the village. Due to the numerous constraints on decision making in the actual village, the long-established nature of many of the heuristics and customs, and the by-and-large risk-averse nature of the population, there is relatively limited decision-making diversity in the village. Consequently, we deemed this assumption to be reasonable.

**C3. Learning.** Agents can adapt their behaviour in the model in response to changes in circumstances and as their memory of the past evolves, but they do not learn *per se*.

**C4. Individual sensing.** Agents are assumed to have knowledge of past crop yields and current produce prices which aids them when making decisions about their future crop mix and in forecasting their future finances. This forecasting process is also aided by finance controllers having knowledge of the possible future circumstances of the agents that they are responsible for.

**C5. Individual prediction.** Forecasts are used in a couple of important ways in the model. Firstly, when updating their crop strategy, households make forecasts about future crop yields and about their grain requirements for the coming year (see *S6*). Secondly, finance controllers regularly make twelve-month income and expenditure forecasts in order to assess whether they can afford to make immediate debt repayments or buy meat (see *S11*).

**C6. Interaction.** Households do not interact with one another, and nor do the animals, fields, or polytunnels. Villagers, however, can indirectly affect one another's circumstances through their influence on their household or finance controller. This influence is primarily, though not exclusively, financial in nature.

**C7. Collectives.** There are two main types of collective in the model. Firstly, there are households. Households consist of a collection of villagers, as well as fields, animals, and other assets, and they are responsible for certain group-level decisions such as determining crop strategies. In terms of their implementation in the model, households are notably depicted not only as collections of villagers and assets, but also agents in their own right. Secondly, villagers are allocated to finance controllers who manage finances on their behalf. When multiple villagers are allocated to a given finance controller, they essentially become a financial collective. These financial collectives can be the same as a household or they can be a subset of household members. They are not formal social units in Namsa, but they help approximate how real-world finances are managed.

**C8. Heterogeneity.** There is scope for very substantial agent heterogeneity in the model. Villagers in particular have a wide range of state variables which in combination produce circumstances that can be quite particular to them. Households can also differ substantially as a result of differences in members and in assets. There is somewhat less heterogeneity when it comes to animals as only age, sex, and ownership can differ. Fields, meanwhile, are only distinguished by their ownership, their usage, and whether they can support rice cultivation.

**C9. Stochasticity.** The model has a large number of stochastic components. These are used in the initialisation stage to generate diversity in the initial simulation conditions and also to “reproduce variability in processes for which it is unimportant to model the actual causes of the variability” ([8], p. 2765). An example of the former is the stochastic determination of animal ages, as well as goat and chicken sexes, at initialisation. An example of the latter is the stochastic determination of a villager's career pathway when career junctures are reached.

**C10. Observation.** Almost all household and villager variables are logged at each timestep to allow in depth post-simulation diagnostics to be performed. This is done so that analysis need not be constrained by prior assumptions about what is of interest. Important events are also logged, allowing the impact of specific stressor events on agent states to be explored.

## S. Sub-Models

**Fig. 3:**
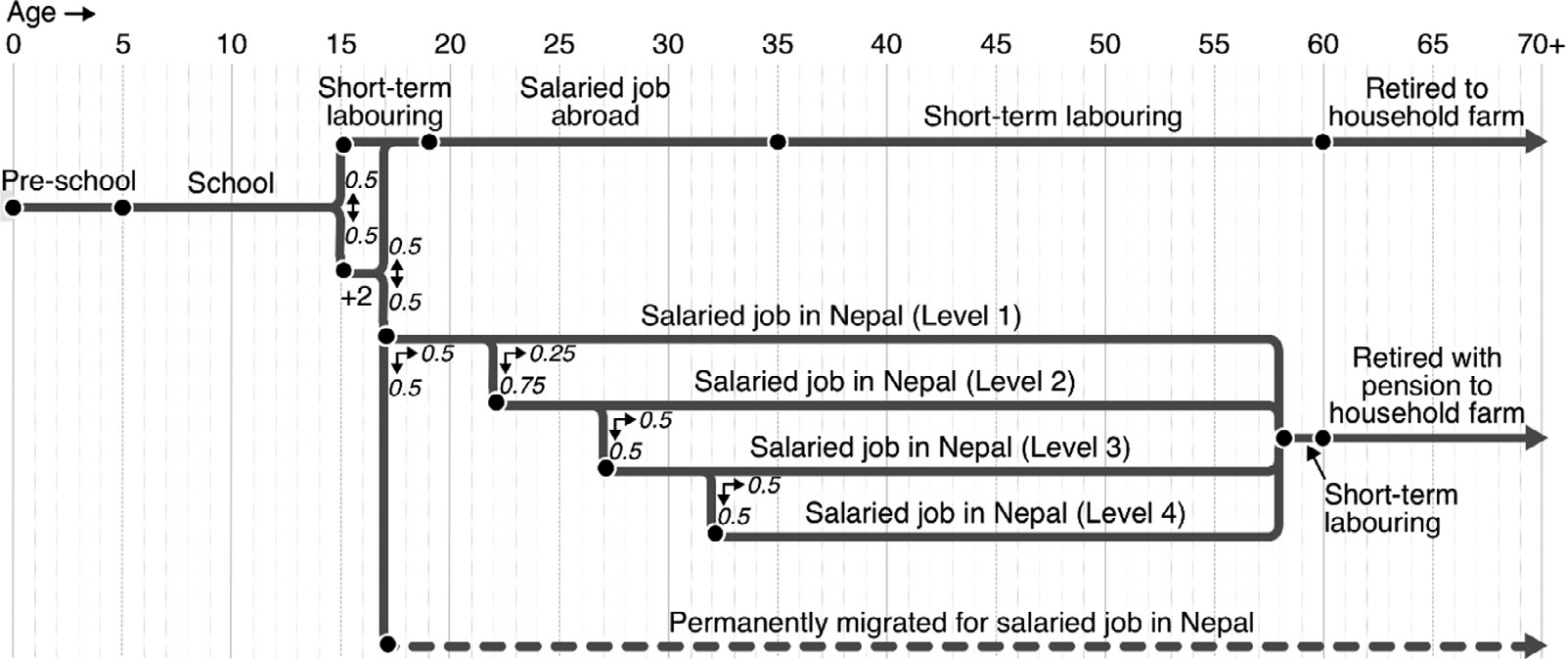
Stochastic flow diagram showing the range of potential life-courses of male villagers. Nodes represent change points, while arrows show the probability of travel along different branches.

**Fig. 4:**
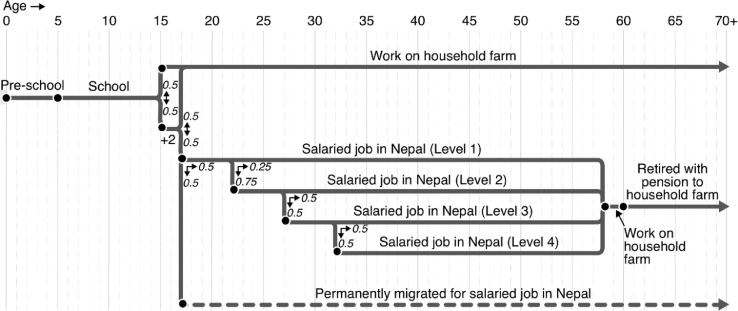
Stochastic flow diagram showing the range of potential life-courses of female villagers. Nodes represent change points, while arrows show the probability of travel along different branches.

**S1. To update career.** The stochastic flow diagrams shown in Fig. 3 and Fig. 4 illustrate the life stages that the villagers in the model progress through as they age. They are based on the contemporary expectations of the villagers in Namsa as discussed in the fieldwork focus groups [24]. The probabilities outlined in the diagram represent the best guesses of the young persons’ and typical life focus groups, with their choices being informed by recent community experience.

In most cases, the life-course change points in the model fall on specific birthdays.^7^ However, education represents an exception. For a villager to begin or complete school, or to begin or complete +2 (i.e. college), they must meet the age condition shown in the flow diagram on the 100^th^ days of the year – the approximate start of the Nepalese school calendar. When villagers complete school or +2, their educational attainment variable is updated to reflect this. Similarly, when villagers transition to a new stage in their life-course, their career variable is updated to reflect this.

**S2. To calculate food expenses.** The basic daily food requirement of an adult female household member is set out in Table 5. These values were determined during the 2017 fieldwork visit through consultation with two of the women who cook for their families on a daily basis [24]. They measured out combinations of ingredients that they adjudged to constitute a typical meal for one female adult. These ingredients were then weighed. Variation in meal types over the course of a typical year is taken into account in the figures. In the case of non-adult females, each foodstuff value in Table 5 is multiplied by the applicable conversion value set out in Table 6. These conversion values are based on data from the Ministry of Agricultural Development [14] on age-sex consumption differences. Household members who have salaried jobs abroad or who have emigrated do not incur food expenses in the model. Household members who are engaged in short-term labouring have their food needs reduced by a half on the days that they take up such opportunities. Similarly, household members who have salaried jobs in Nepal or who attend school or college have their food needs reduced by half on the days that they work/study^8^ because they typically consume one of their main meals outside of the village on these days. In addition to their regular food consumption, villagers may consume up to three portions of meat per week (see *S11*). The particular number depends on the weekly meat consumption variable of their finance controller. Meat consumption is paid for each Sunday. A notable assumption is that market prices for both meat and other foodstuffs will not fluctuate over time. This simplification was made to avoid over complicating the model dynamics – the desired focus of the model at this stage is on the stressors outlined in *Sc*, of which food price fluctuations is not one.

**Table 5:** Daily food requirements of an adult female villagers. These values represent daily averages, thus variability over time is accounted for but not explicitly represented.

Foodstuff	Quantity required (kg / day)	Market price (NPR)
Maize	0.026	0.65
Millet	0.026	0.494
Wheat	0.016	0.352
Rice	0.304	6.08
Potato	0.100	2.2
Other foodstuffs (excl. meat)	-	52

**Table 6:** Pre-meat food consumption needs of various age-sex groups relative to a representative adult woman. The need ratios are derived from the recommended calorific consumption figures for each age-sex group [14].

Female		Male	
Age	Food consumption relative to an adult woman	Age	Food consumption relative to an adult woman
16+	1	16+	1.29
13-15	0.93	13-15	1.10
10-12	0.89	10-12	0.98
7-9	0.88	7-9	0.88
4-6	0.76	4-6	0.76
1-3	0.56	1-3	0.56
0	0.37	0	0.37

**S3. To die.** When a villager is scheduled to die, they ask their partner – should they have one – to set their widowed status to true.^9^ If their partner has a future birth scheduled, this is cancelled unless it is set to occur within next nine months. Next, any cash or debts the villager has are transferred to their finance controller, unless they are their own finance controller, in which case their wife (or household, should they not have a wife) will inherit any such cash or debts. Following this, the villager's household incurs funeral expenses of NPR 200,000^10^ and the villager will cease to exist. The expenses figure is based on conversations that the fieldwork wiki authors had with two households that had gone through the funeral process relatively recently [24]. Occasionally, children may be orphaned as a result of a death. If they have paternal grandparents, they will be adopted by those grandparents. Otherwise, they are assumed to be adopted by their maternal grandparents which means they will leave the village and cease to exist in the model. If the deceased was a male finance controller, his wife and children (should he have any) are assumed to move to his wife's village and therefore cease to exist in the model. If the deceased was the sole member of their household, their house, fields, and animals will also cease to exist.

**Fig. 5:**
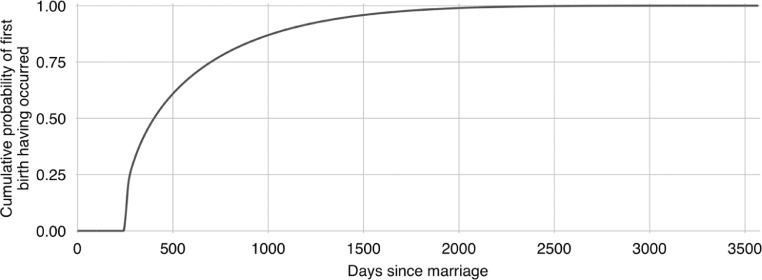
The cumulative probability of first births having taken place with time from marriage. The curve is fitted to values set out in Table 1 of Karkee and Lee [10] for Kaski district in the Mid-Hills of Nepal.

**S4. To marry.** In line with local tradition, when a female villager marries, she will leave the village and cease to exist in the model. In addition to this, her parents will incur an NPR 100,000 expense – the cost for them of providing a dowry. When a male villager who has permanently migrated marries, his parents will similarly incur an NPR 100,000 expense. His role in proceedings will then also be over. When any other male villager marries, a new female villager is created to be his wife. Her current age, her destined age of death, her educational attainment, and her career will be determined using the same methods as those employed in villager initialisation (see *I1, I2, I3* and *I6*). Her household will be the same as her husband's household. Her desired number of children will be stochastically determined with the value being drawn from one of the two fertility scenario distributions (see *Sc2*). And the timing of her first child's birth will similarly be stochastically determined with the value being drawn from the distribution shown in Fig. 5. This distribution is based on data gathered by Karkee and Lee [10]. The marriage ceremonies themselves are assumed to be cost neutral for the households involved as gifts from the guests tend to offset the costs. However, a transfer of wealth from the parents of the bride and groom to the new couple does take place. In the model, the groom's parents (if they are alive) and the bride's parents both gift the newlyweds NPR 100,000. This Fig. represents a typical value according to the fieldwork wiki authors [24] – it varies somewhat in reality.

**Fig. 6:**
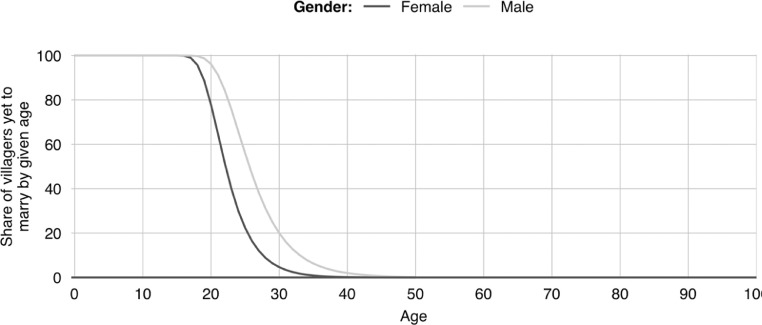
Share of villagers who are yet to marry by age [16].

**Fig. 7:**
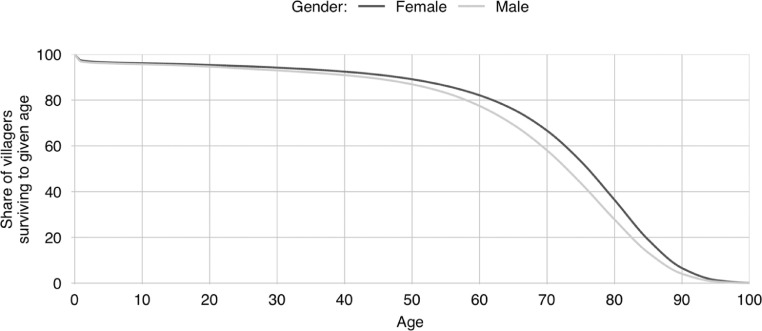
Share of villagers surviving to successive ages in the model [30].

**Fig. 8:**
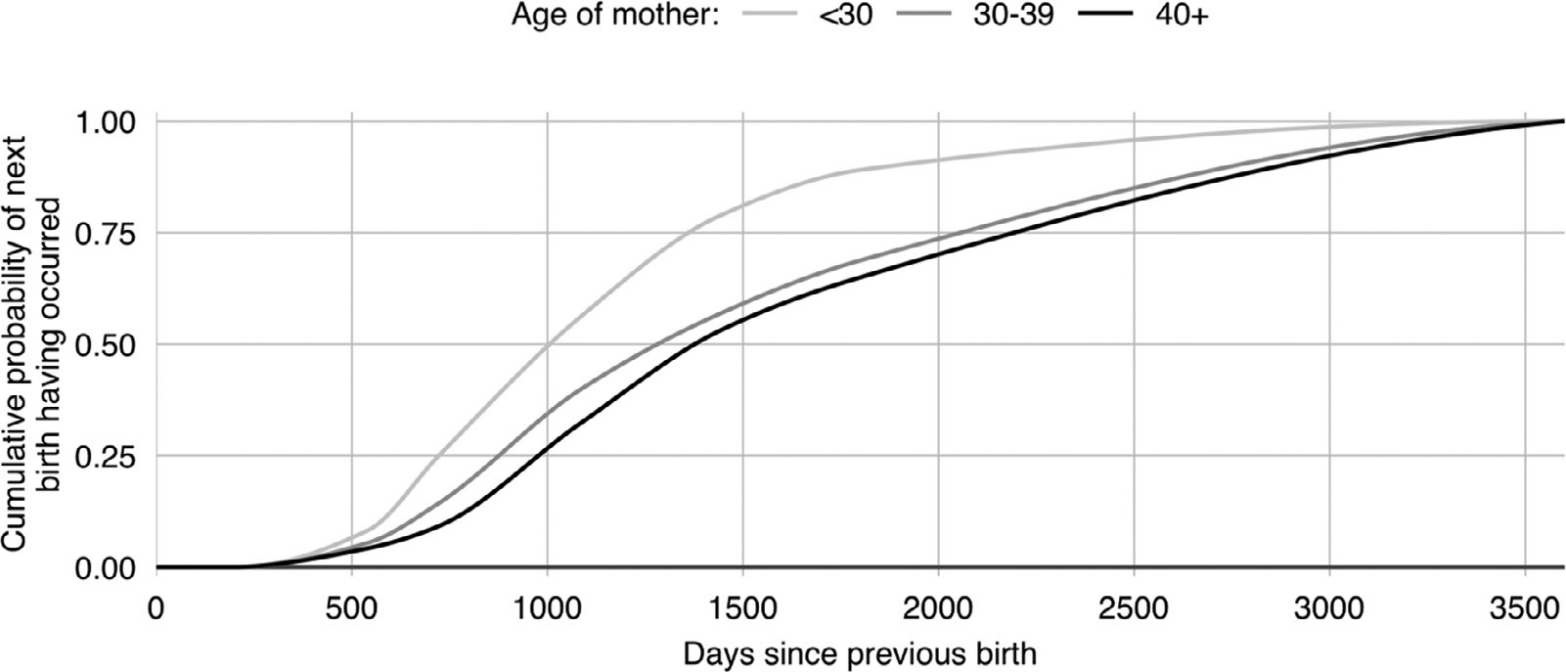
The cumulative probability of a birth taking place with time from the previous birth for mothers who are yet to reach their desired number of children. The curves are fitted to values set out in Table 2 of Karkee and Lee [10] for Kaski district in the Mid-Hills of Nepal.

**S5. To give birth.** When a villager gives birth, a new villager is created. Its age is set to zero, its education is set to pre-school. Its parents are set to the villager who has given birth and to the partner of that villager. Its sex is randomly determined with an equal chance of it being either male or female; more nuanced genders and sexualities are not modelled as information was not forthcoming during the field survey. Its destined age of marriage is determined by drawing a value at random from the sex appropriate marriage age probability distributions that are associated with Fig. 6. These probability distributions were derived from the Nepal Demographic and Health Survey [15]. The child's destined age of death is similarly determined by drawing a value at random from the sex appropriate mortality age probability distribution that is associated with Fig. 7. The mortality probability distributions were derived from the 2015 life table for Nepal [30]. Once the new villager has been initialised, its parents update their list of children to include the new villager. If the mother's child count is still below her desired number of children, another birth will be scheduled. The days till this birth will be stochastically determined with the value being drawn from the distribution shown in Fig. 8. These latter distributions are based on field data gathered by Karkee and Lee [10].

**S6. To update crop strategy.** This is a multi-step process. Firstly, households conservatively update their forecast of future grain needs, which means assessing the expected grain consumption of household members, livestock, and poultry for the year ahead (see *P2-6* and *S2*). Secondly, they forecast per-field yields for the coming year for each crop. This is done by applying exponential smoothing to yield data from the previous ten years.^11^ Following this, an estimated minimum number of fields that need to be allocated to maize, millet, and wheat in order to fulfil the household's grain needs during the forthcoming year can be calculated.^12^ Next, the remaining fields – minus the specialist paddy fields – will be divided between potato, cabbage, and cauliflower production using a fixed ratio of 20:9:3. This ratio represents the standard ratio for cash crops as revealed by the fieldwork household survey [24]. Fields are non-divisible so, when rounding, potato is prioritised over cabbage and cauliflower, and cabbage is prioritised over cauliflower. Paddy fields are always allocated to rice production. The number of fields that are allocated to each crop during this process represents the household's current crop strategy. The way that crop strategies are modelled is one of the bigger simplifications in the ABM. The processes outlined above constitute a rough generalisation of household behaviour when it comes to crops. In the actual village, crop mix decisions were made less deterministically, with the decision process differing slightly between households.

**S7. To plant crops.** When plantation occurs, the status of the chosen fields is updated to reflect their new usage. Households then incur a cost equal to the seeds required per half ropani for that crop, multiplied by the current market price of the crop, multiplied by the number of fields to be grown, plus the per half ropani cost of fertiliser, multiplied by the number of fields to be grown (these values are shown in Table 4). Cabbage and cauliflower represent slight exceptions to this rule as cabbage and cauliflower seeds have a fixed price of NPR 300 per ropani. Households without male cattle or buffalo will also incur a cost of NPR 425 per field for hiring oxen to plough their land.^13^

**S8. To harvest crops.** If the current day is a harvesting day, households receive income from the sale of the relevant crop. The income is equal to the market price of the crop, multiplied by their total crop yield. Market prices are fixed in the model at the rates shown in Table 4, but yields have a degree of variability (see *Sc3*). For households without male cattle or buffalo, harvests also result in an oxen hire cost of NPR 425 per field that the relevant crop is planted in. In the actual village, households will often exchange labour when engaging in agricultural tasks. Sometimes this involves money exchanging hands, but we have decided not to explicitly simulate the hiring of agricultural labour in this version of the model as it would require a number of additional assumptions be made. Another notable simplification in this part of the model is the assumption that harvests are always sold in their entirety. In Namsa, only the cash crops are typically sold. However, by selling harvests in the model and then buying produce as required, management of household food stocks is greatly simplified. It is assumed that the grain (i.e. the non-cash crop produce) is both sold and bought at the fixed market price given in Table 4, so households are typically not financially advantaged or disadvantaged by this buy-back model.

**S9. To perform household fission.** Prior to the 2015 Nepal earthquake, when there was more than one son in a household the elder son(s) traditionally claimed a share of their family's land and built their own home around the time that they married and had their first child. However, following the earthquake – which occurred towards the end of the initial data gathering phase – the cost of construction jumped significantly, forcing a delay in household fission.^14^ In the model, it is assumed that the elder sons of the referent who have not permanently migrated will still build new homes on the portion of the family's land that they are entitled to inherit, but only after (a) they have married and (b) they have enough money to afford to build the home. It is assumed that the new home will cost NPR 625,000. This is a mid-level Fig. for what households were forecasting new homes to cost when we returned in 2017. As soon as the couple collectively have this sum of money, plus a buffer of NPR 35,000, they will establish a new household. This means, firstly, claiming the share of the parent household's land that the son is entitled to. This entitlement is calculated using the following equation, with the result being rounded to the nearest half ropani:

$$E\;=\;\frac{F}{S+1}$$

Here, E is the departing son's field entitlement, F is the parent household's current field count, and S is the number of sons the referent has who are still members of the household at that moment in time (this includes the departing son). If the entitlement is below six ropani, the son will migrate with his wife and children instead of forming a new household as the entitlement would be insufficient for an economically viable farm. If the entitlement is six ropani or more, the son will immediately claim any fields from the parent household that are not currently in use and will progressively claim additional fields from his parent's household as they are harvested until he has his full entitlement. In the model, it is assumed that parent households will always retain any paddy fields that they own, so only regular fields will be transferred. The members of the new household will be the departing son, his wife, plus any children they may have. The household's cash stock will be set to the couple's remaining personal cash stock after the cost of building the new home is deducted. The household will purchase poultry and livestock using the method outlined in *S10*. The youngest remaining son in a household will ultimately inherit his parents’ home and remaining land unless he chooses to migrate. Consequently, he will not need to engage in household fission himself.

**Fig. 9:**
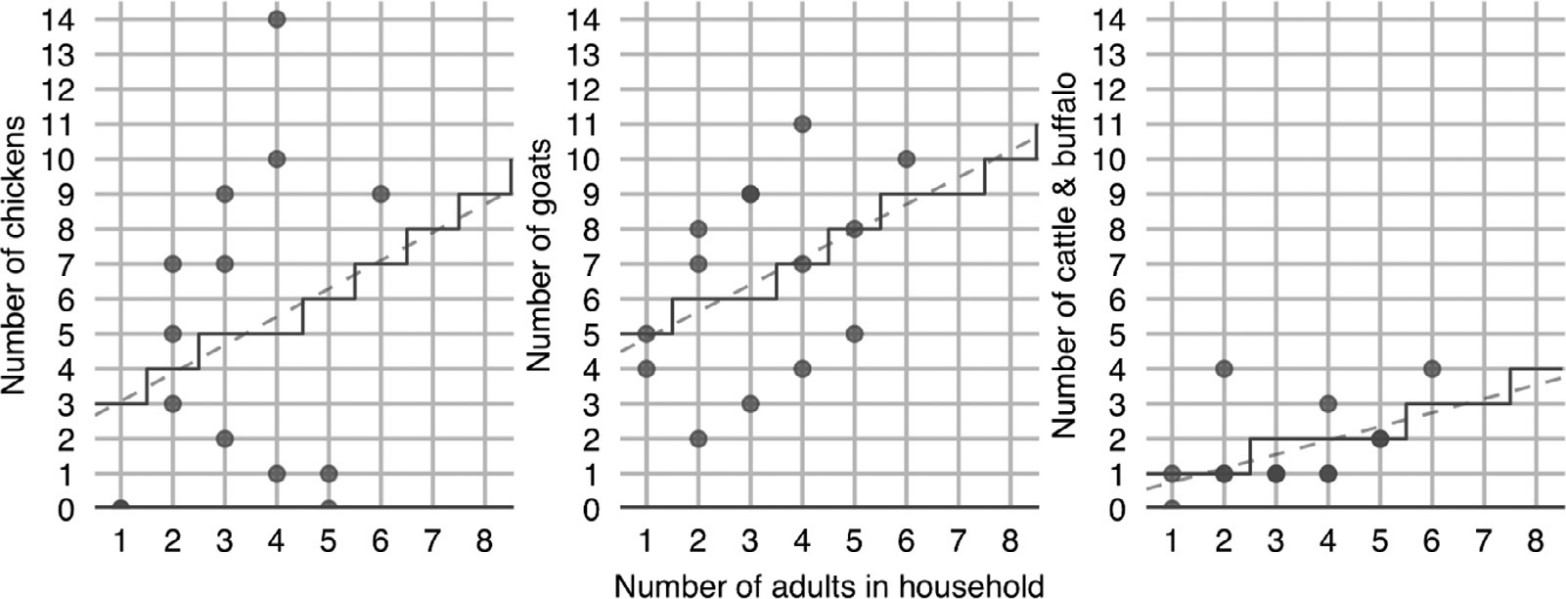
The stepped line shows the deterministic relationship between the number of adult members a household has and the number of each animal type it owns. The grey points show the actual data from Namsa, while the dashed line shows the linear regression line.

**S10. To determine animal ownership.** During the fieldwork decision-making focus group [24], the main factor determining livestock and poultry ownership was said to be the number of adults available to engage in animal husbandry.^15^ Consequently, in the model, animal ownership is deterministically linked to the number of adult household members (>18 years old), as shown in Fig. 9. The relationship model used for each type of animal is based on linear regressions which were estimated from the Namsa data [24]. While it is clear from Fig. 9 that the real-world determinants of chicken ownership are more complicated than this, linear regression provides a practical simplification for the purposes of model implementation. Importantly, there are a handful of cases in respect to determining bovine ownership in which caveats apply. If households have less than 5.5 ropani, they are deemed incapable of supporting any bovine animals as they will not be able to produce sufficient crop fodder to support any cattle or buffalo. For related reasons, households with 5.5 to 10.5 ropani will have their potential bovine ownership capped at one, households with 11 to 16 ropani will be capped at two, and households with 16.5 to 21.5 ropani will be capped at three. These rules are based on discussions in the fieldwork resource flows focus group [24], although they represent a simplification of reality. Poultry and livestock ownership are less deterministically defined in the actual village, with animal preferences and strategies varying somewhat.

When the number of animals a household has is below the number it should have given its size and land, the household will purchase additional animals to correct the disparity. Chickens are bought when 14 days old at a cost of NPR 400. Their sex is randomly assigned. Goats are bought when 112 days old at a cost of NPR 5,000 and also have their sex randomly assigned. If the new animal is to be a bovine, it will be bought when 548 days old at a cost of NPR 300 if it is an ox or cow, or NPR 10,000 if it is a buffalo. The breed is stochastically determined with two fifths of bovine designated buffalo, while the remainder are designated cattle. If the household does not already have buffalo or cattle, the animal will be designated female, otherwise it will be designated male. These rules approximate the livestock sex and breed preferences observed in the Namsa fieldwork survey data [24]. The purchase cost and age values are based on those reported by the fieldwork wiki authors [24]. If the number of animals a household has is above rather than below the number it should have given its size and land, the household will instead sell animals to correct the disparity. Animals are sold in order of age, from the youngest to the oldest. The money the household receives when selling an animal depends on the animal's breed and age. It is assumed that an animal's worth increases linearly between the time it is purchased and the time it reaches slaughter age.

**S11. To assess finances.** Once all income and outgoings have been determined for the households and villagers, finance controllers take stock of the impact the transactions have had on their cash situation. If the transactions during the current time-step have resulted in a finance controller having a negative cash balance, the controller will typically need to take out a loan to cover the shortfall.^16^ This loan is added to the sum of any existing debt the controller may already have. An exception is made if the finance controller is a household and if that household has another finance controller among its members (i.e. a financially independent son). In such cases the member will cover the household's shortfall if he has sufficient cash, thus helping them avoid accumulating debt. This kind of intra-familial support is fairly typical in Namsa according to the fieldwork decision-making focus group [24]. Next, if a household has debts, the daily interest due on that debt is calculated and added to the total. The annualised interest rate is set at 20% which is typical for the area according to the fieldwork wiki authors [24]. Following this, should the time-step correspond to the first day of a month, finance controllers will conservatively forecast their income and expenditures over the coming year, and the effect these will have on their cash situation. This will enable them to assess whether they can afford to make immediate debt repayments (should they have debts) or afford to buy meat during the coming month without getting into a negative cash situation later in the year. Debt repayments are prioritised over the purchase of meat so the feasibility of these is assessed first. If a household or villager has debt and its cash forecast is in the black for the entirety of the coming year, it will calculate what its cash balance is forecast to be at its lowest. If the household or the villager's debt is less than this amount, it will immediately pay the debt off in full, otherwise it will simply pay down the debt by the forecast minimum cash balance. In the scenarios in which earthquakes occur, households will be issued with reconstruction loans which are treated separately from any other debt they may have. The repayment of a reconstruction loan is only contemplated when households are free of other debt. Repayments are made in monthly instalments, each of which amounts to one sixtieth of the original loan. Households only make these monthly repayments when their forecasts suggest they can afford to do so without getting into a negative cash situation later in the year. The reconstruction loans are interest free. If a finance controller has cash remaining after paying off any debts it may have had and after meeting any loan repayment obligations, it will consider how many portions of meat it can afford to buy for its dependents each week for the coming year while still meeting any reconstruction loan repayment obligations it may have. The cost of providing meat to a villager is NPR 102 multiplied by the villager's food consumption multiplier (see Table 6). Up to three portions of meat per week are considered. Once the maximum viable number of portions that the controller can afford has been determined, the controller's weekly meat consumption variable will be set to reflect it. Of course, this representation of how households manage their finances incorporates a number of simplifying assumptions. The reality, which was discussed during the fieldwork decision-making focus group [24], can be more complicated and varies somewhat between households.

## I. Initialisation

For the model to provide useful insights into the future evolution of villages like Namsa, the initial model conditions need to be realistic. One option would have been to directly replicate the fieldwork village in virtual form. However, there are two significant ethical reasons why this would be inappropriate. Firstly, doing so would pose a privacy threat to the research subjects as, in combination, the individual and household attributes that would be included in the code could potentially lead to their identification by third parties [6]. Secondly, the act of simulating the behaviour and life course of actual individuals might reasonably be perceived as infringing on the dignity of the research subjects. For example, few of the villagers would, presumably, appreciate their deaths being simulated. For these reasons, it is necessary to either obfuscate the empirical data prior to its use or to use synthetic datasets in the model *in lieu* of the empirical data.

There are a number of obfuscation techniques available such as randomization, data swapping, and data desensitisation^17^ [1]. However, their use in this case would be complicated by the existence of numerous dependencies between data points and the need to retain data granularity and a consistently high degree of verisimilitude. Furthermore, most obfuscation techniques focus on details rather than structures, so they could potentially leave signature patterns within datasets that could still enable re-identification.

The alternative approach, population synthesis, has seen a flurry of interest in recent years as it represents a key stage in the spatial microsimulation process [13]. Typically, spatial microsimulation requires a population of synthetic individuals be generated in instances where aggregate statistics are available, but individual level data is restricted to a subset of the real population.^18^ While there are a range of methods available for population synthesis, most of them essentially involve combining the aggregate information with the individual level data that is available to construct a synthetic population which approximates the “correlation structure of the true population” ([2], p. 266). The strengths and weaknesses of each method of population synthesis can, however, differ substantially.^19^ Importantly in our case, only a fraction of the techniques perform well in creating realistic household groupings of synthetic individuals. One such example is given by Gargiulo et al. [5] who set out a sample-free method for generating a synthetic population organised in households. Their approach respects a variety of statistical constraints such as “distribution of household types, sizes, age of household head, difference of age between partners and among parents and children” [5] – all of which is a requirement for the model set out here. However, like most population synthesis methods, the approach used by Gargiulo et al. [5] assumes a significantly larger initial dataset can be drawn upon than the one available in our case and it was also designed with the generation of rather larger populations in mind than the one we require. Unfortunately, their method does not downscale particularly well when it comes to the use of small input datasets and the need for small population outputs. However, approaches such as that of Gargiulo et al. [5] do offer a framework from which we can take inspiration.

**I1. Population synthesis.** Given the limitations of existing methods of population synthesis, it has been necessary to design a bespoke approach – an approach that can generate realistic households composed of realistic individuals, and that can approximate the composition of household types seen at the fieldsite using just the data we have available. Rather than generating a population of individuals and then allocating them to households (the approach typically used), we determine a set of household types and then generate individual household members iteratively to suit the types of household created. The main steps are as follows:

1. The number of households that should be generated is specified. For the purposes of this study, this will always be 14 – the number of households that resided in Namsa at the time of the fieldwork [24].
2. Each of the households to be generated is assigned a household type from the options shown in Fig. 10. Assignment is done through the use of stochastic universal sampling (SUS) to ensure that a minimum spread of different household types is chosen while still allowing for a degree of variability in each model run. The probability of each household type, P(t), being selected is proportionate to the observed occurrence of that household type during the main fieldwork phase in 2015.

**Fig. 10:**
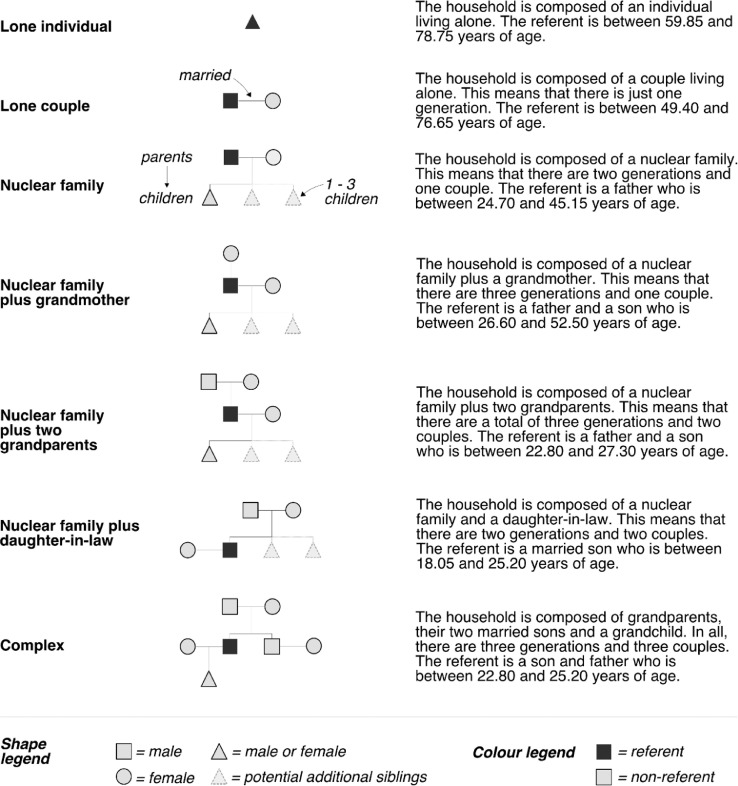
The seven basic household structures observed in Namsa in 2015. They form the basic household types that are generated in the population synthesis process. This data comes from the fieldwork household surveys [24].
3. The next sequence of steps involves populating each household in turn with members. This process starts by selecting the age of the household's referent individual, $a_{r}$. These are the individuals shown in black in Fig. 10. The age is selected using a simple random draw from the set of observed ages of the equivalent individuals in the equivalent household types in Namsa, $P(a_{r}|t)$. A small amount of noise is then added or subtracted from this Fig. (±5%). This ensures that referents will not all share the same birthday and it adds diversity to the potential outcomes that goes beyond the observed referent age values. Also determined at this point is the sex of the referent. If t is not a lone individual, the referent is always male. However, if t is a lone individual, the sex is instead randomly determined – the probability of the referent being male or female is assumed to be equal. In the case of lone individual household types, the household will be fully populated at this point so the process of populating the next household in the list will then commence. If the household is not a lone individual, the next stage of the process commences.
4. The next step is to select the age of the referent's partner, $a_{p}$. This involves adding the result of a simple random draw from the set of observed age differences between husbands and their spouses, to the previously selected $a_{r}$value. Importantly, any of the observed age differences that would result in the partner being younger than 18 years-old^20^ are excluded from consideration and a small amount of noise (equivalent to ±0.5 years-of-age) is added to each observed value before the draw is made. The noise is applied so that the birthday of the partner will usually differ from that of the referent. The noise value is kept small so as not to radically alter the initially determined value. The sex of the partner is then set to female. After this, the number of children the couple will have during their lifetime is decided probabilistically according to $P(c|a_{p})$. These probabilities are again based on the fieldwork survey data from Namsa [24]. If the household is to be a lone couple, the process of populating the next household in the list will then commence. For the remaining household types, the process continues.
5. If the household type is not a nuclear family plus daughter-in-law, the age and sex of the referent's current children is decided, and the provisional date of the next birth is selected if the current number of children is not equal to the value of the lifetime number of children that was determined in the previous step. This process involves first deciding the age of the eldest child probabilistically based on $P(a_{c1}a_{p})$, and then selecting the birth interval to each subsequent child probabilistically based on P(birthInt). Both of these probabilities are again based on the data gathered during the surveys [24]. If an interval yields a future date, that value will represent the provisional date of the next birth. As the birth interval values are not integer years, it is not necessary to add noise to the results. Following these steps, the sex of each child that has been born is randomly determined – the probability of each child being male or female is assumed to be equal. If the household is to be a nuclear family, the process of populating the next household in the list will then commence. For the remaining household types, the process continues.
6. The next step is to select the age of the referent's mother, $a_{m}$. This involves adding the result of a simple random draw from the set of observed age differences between referents and their mothers, to the $a_{r}$ value. Again, a small amount of noise (equivalent to ±0.5 years-of-age) is added to the result. The sex of the referent's mother is then set to female. If the household is to be a nuclear household plus grandmother, the process of populating the next household in the list will then commence. For the remaining household types, the process continues.
7. Next, the age of the referent's father, $a_{f}$, is determined. A simple random draw is made from the set of observed age differences between referents’ mothers and referents’ fathers with the chosen value then added to $a_{m}$. A small amount of noise (equivalent to ±0.5 years-of-age) is again added. The sex of the referent's father is set to male. If the household is to be a nuclear household plus grandparents, the process of populating the next household in the list will then commence. For the remaining household types, the process continues.
8. If the household is a nuclear household plus daughter-in-law, the referent will not have children, but he may still be living with siblings. The number of children the referent's parents have is decided probabilistically according to $P(c|a_{m})$. This probability is again derived from the household surveys [24]. As the referent is the only one of the siblings who will be married at the initialisation point, he is assumed to be the eldest. The interval between each sibling in age is also determined probabilistically by repeat picking based on P(birthInt). Following these steps, the sex of each sibling other than the referent is randomly determined. After this, the process of populating the next household in the list will commence.
9. At this point, just one household type remains to be completed: the *complex* household. In this instance, the age of the referent's brother, $a_{b}$, is chosen next based on P(birthInt). As reasonable age values for $a_{b}$ are bounded in this case by the need for both brothers to be relatively close in age (less than 3 years), only a subset of the potential birth intervals is included in the possibility set. The age difference which is selected is added to $a_{r}$. Next, the age of the brother's wife, $a_{sil}$, is chosen by selecting a value using the same process as that used for determining the age of the referent's partner. The sex of the referent's brother is set to male and the sex of the referent's sister-in-law is set to female. After this, process of populating the next household in the list will commence.

This basic flow is repeated until all of the households have been populated. This bespoke approach allows creation of virtual villages that should be qualitatively similar to Namsa in terms of population and household structure, while preserving the anonymity of the research subjects. It also means that actual individuals are not simulated, so it addresses the core ethical concerns mentioned earlier. Furthermore, it is well suited to the data available and can create small populations without issue. There are, however, certain limitations to the approach. Firstly, the archetypal households are limited to those observed at the fieldsite in 2015 and, secondly, the reference distributions that inform the probabilistic selections are based on small samples. This means that possible outcomes are always quite strongly tethered to the particularities observed in Namsa.

**I2. Initialise villager educational attainment.** Villagers who are less than 2,090 days old will be in pre-school at the time of model initialisation as they will have been less than five years old when the current school year started. Villagers who are older than 2,090 days but younger than 5,740 days will be in school as they will have been between five and 15 years old when the current school year started. Villagers who are older than 5,740 days but younger than 6,570 days will either be undertaking their +2 (i.e. college) or will have left the education system as they will have been between 15 and 18 years old when the current school year started. Both possibilities are equally likely. Of the villagers who are older than 6,570 days, but under 28 years-of-age, by default a quarter will have attained +2 qualifications, a quarter will have left education after completing their SLC (i.e. the final examination in the Nepalese secondary school system), and the remaining half will have left school before attaining any qualifications. Villagers who are over 28 years-of-age but under 45 years-of-age will have a 25% chance of having attained their SLC. The remainder – along with villagers who are over 45 years-of-age – will have left school before attaining any qualification. These rules have been derived from the data on educational attainment that was collected during the fieldwork household surveys and from discussions during the typical life and young persons’ focus groups [24].

**I3. Initialise villager career.** Villagers who have completed their education will be allocated to a career pathway at initialisation. The particular career pathway that is selected for a villager is determined using the same principles employed in *S1*, with the villager's sex, age, and educational attainment all being taken into account.

**I4. Initialise villager age of marriage.** The marriage age of those who are single at the time of model initialisation is determined by drawing a value at random from the viable values^21^ in the sex appropriate marriage age probability distributions that are associated with Fig. 6. A similar process is used for women who are already married at initialisation, except the age of their eldest child (should they have one) is taken into account in determining what is a viable value as it is assumed that women will be married prior to having their first child. The marriage age of husbands is then determined based on the values assigned to the women.

**I5. Initialise villager fertility.** The desired child count of married women who are under the age of 50 is also determined at initialisation. The process used to select this value is the same as the one outline in *S4*, except it takes into account the number of children the women already have, the time since they had their last child, and the time since they married. Specifically, the number of children that a woman already has determines her minimum desired fertility, women who have been married more than 9.78 years without having a child are assumed to desire no children, and women whose last child was born 9.86 years ago are assumed to desire no more children. The choice of these values is based on data from the Nepal Demographic and Health Survey [15]. Women who desire more children than they currently have will also have the timing of their next birth determined at initialisation. This is done using the same method as that outline in *S4* for women who are yet to have a child, and *S5* for women who already have at least one child, except the viable values that are drawn from are constrained by the time that has elapsed since the women were deemed to have last given birth or married.

**I6. Initialise villager age of death.** The age at which the newly initialised villagers are set to die is determined by drawing a value at random from the viable values^22^ in the sex appropriate mortality age probability distributions that are associated with Fig. 7.

**I7. Initialise household fields.** The number of fields a household is assigned is determined using a stochastic function that takes into account the number of adults in the household. The function is based on a linear regression model which was fitted to data from the Namsa household surveys [24]. Noise terms derived from the standard error values of the regression model are added to the function to mimic the observed variability between households. The function is as follows:

$$r=\;1.8854+\left(2.0284*a\right)+\eta_{1}+\;\eta_{2}$$

Here, $\eta_{1}\;∼\;N\left(0,\;1.6715\right)$, $\eta_{2}\;∼\;N\left(0.4728\right)$, r being the number of ropani, and a being the number of adults in the household. Values generated by the function are rounded to the nearest half ropani as a half ropani is the standard unit of land used in the model. A floor of five ropani is enforced as this was said to be the minimum viable size of a functional farm.

**I8. Initialise household paddy fields.** Two, three, or four of the fields that are assigned to households with 16+ ropani of land will be deemed suitable for rice cultivation when the model is initialised. The precise number of fields allocated is randomly determined with each option being equally likely.

**I9. Initialise household polytunnels.** Three randomly chosen households who have at least two adults under the age of 60 will be allocated polytunnels. The particular number of polytunnels each household is allocated is randomly determined but will be either one, two, or three. These rules are designed to allocate polytunnels to the kinds of households that had been targeted for support by an international non-governmental organisation (INGO) that ran a tomato cultivation project in the actual village.

**I10. Initialise household animals.** The number and sex of animals each household has at initialisation is determined using the same deterministic method as that outlined in *S10*. However, rather than the animals being assigned the default age values for new livestock and poultry, their initialisation ages are randomly determined out of the viable age values.

**I11. Initialise finances.** Finance controllers have their finances initialised using a two-step process. Firstly, they conservatively forecast their income and outgoings for the forthcoming year, while assuming meat consumption of two portions per week and the realisation of expected crop yields (the latter being of relevance only to household). Based on this assessment, the amount of initial cash that they require to meet their consumption and livelihood needs during the coming year without going into debt can be determined. They are then allocated this sum. In addition to this, households may be allocated historical savings or debts. This is done by randomly assigning each household the net finances of one of the households in Namsa (as established in the household surveys), using a sampling without replacement approach. Two households are also assigned a monthly remittance income of NPR 10,000. Finance controllers who are villagers will, meanwhile, be allocated historical savings that take into account their estimated earnings and expenditures since they left education. This is important as it can affect the timing of household fission.

**I12. Initialise past and future yields.** Ten years of historical crop yield data is generated at initialisation to enable the crop strategy selection procedure to function. This is done by repeating ten times for each crop the procedure that is outlined in *Sc3* for stochastically determining a given crop's yield.

**I13. Initialise future yields.** For simplicity, the yield of each crop for each simulation year is predetermined. This is done in the same way as the above procedure, but for fifteen years’ worth of yields instead of ten (simulations are fifteen years long).

**I14. Initialise crop status.** The initial crop strategy of households is determined using the same method as that outlined in *S6*. Wheat, cabbages, and cauliflower are scheduled to be planted at the time the model starts so households assign the applicable number of fields to each of these crop types, following essentially the same process as that set out in *S7*.

## Sc. Scenarios

In the study that the model was originally designed for [24,26], we focused on assessing the potential impact of the 2015 earthquakes, changing fertility rates, and increasing crop yield variability on household finances and village demographics. Two scenario pathways were crafted for each of these stressor types. As explained below, each pathway embodied a certain set of assumptions about how that particular stressor will play out. In Roxburgh [24] and Roxburgh et al. [26] each model run involved simulating one of the earthquake pathways, one of the fertility pathways, and one of the crop variability pathways. This means that there were eight potential pathway combinations in all.

**Sc1. Earthquake scenarios.** The first earthquake pathway is an effort to mimic the impact of the 2015 earthquakes which struck Namsa towards the end of the main fieldwork spell, shortly after the baseline data – which the initial model conditions are generated from – was collected [25]. The details of the scenario are based on the experiences reported by the villagers themselves during the follow up fieldwork in early 2017. Middling values are used where households disagreed about the degree of impact or where experiences differed. The scenario details are as follows:

• Five percent of livestock die as a result of collapsed shelters and stress. Households are not able to sell these deceased animals due to the chaotic circumstances and loss of access to local markets due to landslides blocking the highway.^23^
• There is a fifty percent chance of individuals who live alone leaving the village to reside permanently with family members elsewhere in Nepal. There is also a fifty percent chance of households that are composed of two adults emigrating permanently if one of those adults has a salaried job. When a household leaves the virtual village, their house, fields, and animals cease to exist.
• The yield of the crops that are in the ground between 25 April and 12 June (one month after the 7.3 Mw aftershock) is 30% below what it would otherwise have been due to terrace collapses, soil movements, damage to the village irrigation system, and a reduction in the time that villagers can dedicate to agricultural activities.
• The standard cash crop yield permanently declines by 15% as a result of the reduced irrigation capacity of the village following the damage caused to the village irrigation system which the households are unable to repair.
• Off-farm labouring stops for three months, as villagers focus on dealing with the challenges on their own farm and because of the regional economic disruption. However, once this three-month period is over, off-farm labouring opportunities increase by 50% above the baseline rate for twenty-four months as the recovery and reconstruction processes get underway.
• On 25 October 2016, eighteen months after the main shock, households take out reconstruction loans to fund the rebuilding of their houses. The size of the loan depends on the number of members the household has at that moment in time. Households of one or two members are issued with a loan of NPR 300,000. This rises to NPR 425,000 for households with three to four member, NPR 550,000 for households with five to six members, and NPR 675,000 for households with seven or more members. The role of reconstruction grants is reflected in the chosen loan sizes. Details of the loan repayment process are given in S11.

The second earthquake scenario is a counterfactual in which the earthquakes do not happen and therefore the impacts listed above are not realised. This alternative pathway provides a baseline against which the consequences of the earthquakes for household finances and village demographics can be gauged.

**Fig. 11:**
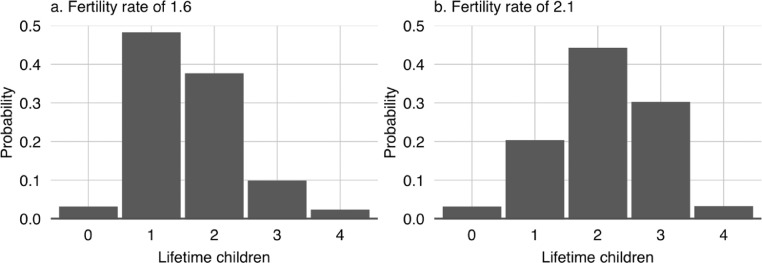
The two fertility rate scenarios decomposed probabilistically by lifetime children.

**Sc2. Fertility scenarios.** The first fertility scenario assumes an average fertility rate of 1.6 children per woman, while the second assumes the slightly higher rate of 2.1 children per woman. Fig. 11 shows how these headline fertility rates translate into probabilities for particular numbers of children in each case. Both of the fertility rates can be considered equally plausible for Namsa over the short- to medium-term based on the discussions during the fieldwork young persons’ focus group Roxburgh [24] and recent demographic reporting for Nepal [16]. Those who attended the focus group – who will represent the next generation of parents – spoke of a clear preference for either one or two children. Taken at face value, this would suggest that a fertility rate of more than one but less than two should be expected. However, a 2014 study estimated that 26.1% of births in the Central development region of Nepal (in which Namsa is located) were unplanned [22]. For this reason, it is assumed that the actual fertility rate is likely to be somewhat above the intended rate. Based on this logic, the chosen fertility rates represent reasonable ‘high’ and ‘low’ scenarios. Notably, the 1.6 rate is equal to the current urban fertility rate, while the 2.1 rate is the Nepalese governments long-term national target [11].

**Fig. 12:**
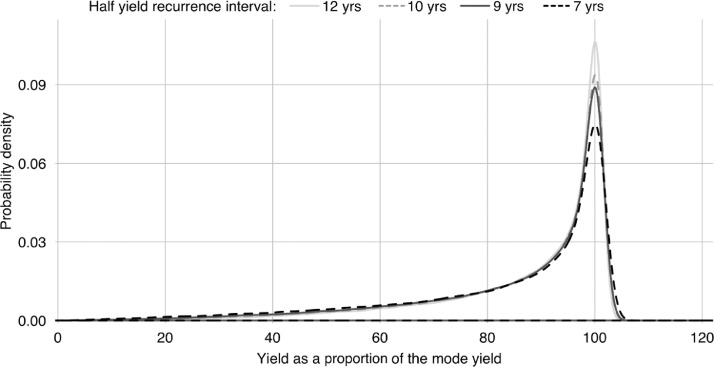
The four crop yield probability curves that are used in the model.

**Sc3. Crop variability scenarios.** The first crop variability scenario assumes a continuation of the status quo in terms of inter-annual yield variability, while the second scenario assumes a slightly higher degree of inter-annual yield variability than is currently reported. This latter scenario is intended to represent the potential consequences of climate change and the increasing threat of pests and diseases for agriculture.^24^ In the model, each crop has a standard (mode) yield as shown in Table 4. When a crop is due to be harvested, this standard yield is multiplied by a number drawn randomly from a crop yield probability function to determine the actual yield. The crop yield probability function that is used depends on the crop type and the scenario that is being simulated. There are four crop yield probability functions in all, each of which is based on a beta function that is calibrated to result in a specific half-yield recurrence interval (see Fig. 12). Half-yield frequencies are used as the concept was readily understood during discussions with the villagers in the fieldwork community history focus group [24]. For simplicity, all of the traditional subsistence crops (maize, millet, wheat, and rice) are assumed to have the same degree of variability in any given run and all of the cash crops (potato, cabbage, cauliflower) are assumed to have the same degree of variability in any given run.^25^ However, the actual yield of a crop in any given year is independent of the yield of the other crops. For subsistence crops, the status quo half-yield recurrence interval is 12-years, dropping to 9-years for the alternative scenario. For cash crops, the status quo half-yield recurrence interval is 10-years, dropping to 7-years for the alternative scenario.

## 3. Determining the necessary number of replicates

**Fig. 13:**
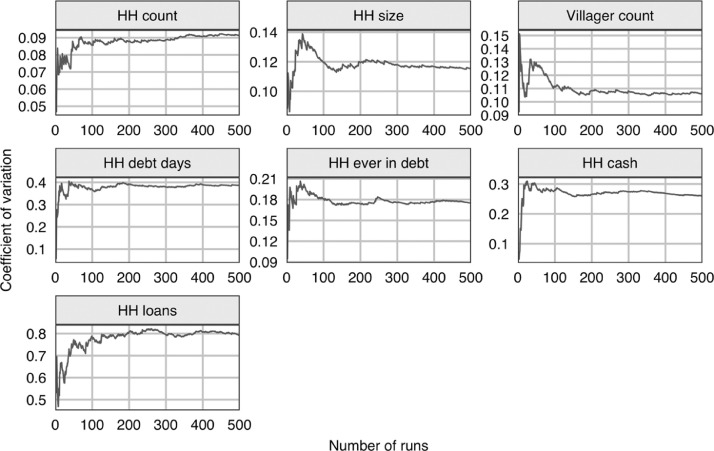
Coefficient of variation of various metrics for increasing numbers of runs in the no-earthquake, low crop variability, 2.1 fertility rate scenario with parameters set to their default values. HH = Household.

Given the stochastic nature of the model, it is necessary to conduct multiple runs for each scenario that is being simulated in order to determine what constitutes typical outcomes and to gauge variability [12]. Following Lee *et al.* [12] and Broeke *et al.* [29], we looked at how the coefficient of variation of various metrics changes with the number of runs – see Fig. 13. In each case, the coefficient of variation largely stabilises after 200 runs. Consequently, this number was considered the minimum number of replicates necessary for the analysis in Roxburgh [24] and Roxburgh et al. [26]. In future studies that use the model, there may be a need to repeat this analysis to take into account changes in the model code or differences in the metrics of interest.

## 4. Model validation

Model validation is the “process of ensuring that there is a correspondence between the implemented model and reality” [31]. It refers to the establishment of legitimacy in respect to the model design and parameterisation decisions [20]. Within the field of agent-based modelling, two broad approaches to validation can be discerned: empirical validation and stakeholder validation. Empirical validation involves assessing how well data produced by a model corresponds to real-world empirical data. This is the most popular approach to validation in the literature and is the “traditional yardstick for establishing confidence in models” ([21], p. 141). However, as Polhill and Salt [21] note, this often requires an infeasible amount of data if it is to be done effectively. It is particularly problematic in cases such as this, where a system has many potential evolutionary pathways, but only one can ever be observed. Stakeholder validation, meanwhile, involves the subjects of the model (or other third-party experts) providing feedback on the appropriateness and accuracy of design decisions [9,18]. In respect to this study, stakeholder validation is more straightforward than empirical validation, but ultimately is reliant on the quality of the judgements that are made by those who are engaged in the validation process.

**Fig. 14:**
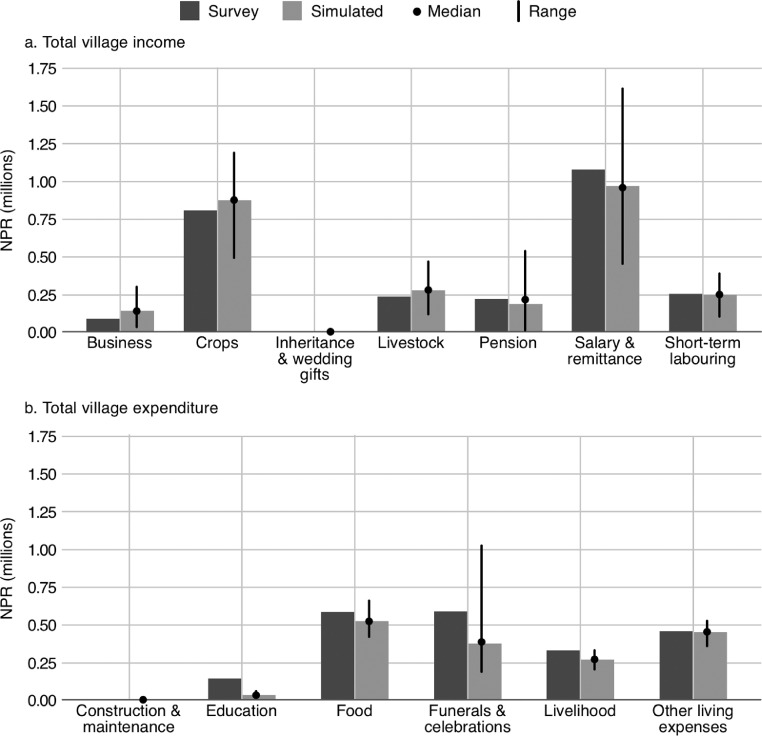
Comparison of surveyed and simulated total village income and expenditure disaggregated by type. Four hundred simulations were conducted in all – 200 with a fertility rate of 1.6 and 200 with a fertility rate of 2.1.

Here, we have drawn upon a combination of empirical validation methods and qualitative validation methods to assess the appropriateness of the model, recognising the respective strengths and drawbacks of each approach. In respect to empirical validation, we conducted four-hundred one-year-long model runs for the no-earthquake, low crop variability scenarios in an effort to approximate the conditions in the year leading up to the fieldwork. We then calculated the total income and expenditure of the village in each replicate, breaking this down by income and expenditure type. We then compared the mean and the range of the values produced in this simulated data to the household survey data [24] for the same categories of income and expenditure as shown in Fig. 14. Before this was done, however, the two datasets were adjusted to account for certain differences in how the model operates compared to real-life. Specifically: (a) Subsistence crops are by and large not monetised in the actual village, so their sale and purchase values are removed from the income and expenditure data in the simulation results; (b) In the model, households are assumed to buy all of their new animals from third parties, while selling all of their new-born animals. In reality, households breed a majority of their own animals so therefore spend less on animals and receive less for animals than the model suggests. To adjust for this, two-thirds of the spending on new animals in the model data is deducted from livestock income (which includes animal sales), with livestock expenditure in turn reduced by two thirds; (c) In reality, households will often consume meat from animals reared within the village rather than purchasing their meat solely from markets as the model assumes. To adjust for this, two-thirds of meat expenditure is deducted from food expenditure and livestock income; (d) Finally, in the actual village, there is a household that owns a grain mill which means it has unusually high business income and livelihood expenditures relative to the other households. It also has anomalous educational expenditure. As the mill is not simulated in the model, these anomalously high income and expenditure values have been corrected for in the benchmark empirical data through the use of mean substitution. In the case of business income and livelihood expenditure, the substitute value was based on the mean per adult business income and livelihood expenditure of other households (i.e. NPR 1,987 and NPR 7,671 per adult respectively). In the case of educational expenditure, the substitute value was based on the mean per child educational expenditure of the other households (i.e. NPR 12,000 per child).

That the survey data points fall within the range of simulated outcomes in most income and expenditure categories is a positive sign.^26^ That said, it is hardly surprising in the case of the short-term labouring and other living expenses categories as they were calibrated to the survey data. It was, however, less inevitable in the case of the other categories as the parameters that underlie them were by and large not derived from the annual income and expenses data gathered during the fieldwork household survey. The values do not exactly coincide and there is substantial variability in a number of the simulated categories, but this is to be expected given the stochastic nature of many of the processes [28]. The only category where the simulated outcomes do not overlap with the survey derived value is education costs. The simulated education values undershoot the reported value by a large margin^27^ – NPR 108,984. The reason for this discrepancy is not, however, clear. It could reflect a problem with the survey data, or an issue with the model design and the parameters which were informed by the fieldwork wiki [24]. Further fieldwork would be needed for the cause to be established.

Whether this comparison of values is of much value is debatable given the number of strong assumptions that were necessary to make the two data sets comparable and the substantial variability in model outcomes. This makes the efforts that were made to cross verify the data that informed the design of the model [24], all the more important. Also valuable was the chance to run the initial ideas for the model by the some of the villagers on our return fieldwork visit in 2017 [24]. This provides confidence that most of the model processes are at least valid “on face” [31]. As an additional step, extensive monitoring of individual runs was conducted to check for unreasonable behaviours [31]. Where unexpected behaviours were identified, investigations were conducted into their cause and corrections were made where appropriate.

## 5. Model sensitivity analysis

Sensitivity analysis (SA) explores the response of a model's outputs to plausible changes in input parameters [12,23]. It is used both to “quantify the uncertainty of the model outcomes that is caused by parameter uncertainties” [29] and to gain insights into model behaviour. Here we use an adapted version of the popular one-parameter-at-a-time (OAT) approach to SA as it is the least computationally demanding of the standard SA methods [29]. This is important because the model is highly computationally demanding. Typically, OAT involves varying one parameter at a time while holding all other parameters at their default baseline values [12]. This enables the impact of each varied parameter on the outputs to be assessed, thus providing some insight into the sensitivity of each output to that parameter and offering a sense of the relative importance of various model components. Here, we deviate from the standard approach to OAT by varying parameters in thematic groups rather than individually. This is done because the model involves an unusually large number of parameters which makes it impractical to examine each one in isolation. The impact of the parameter changes will likely be bigger as result of varying them in groups, than if they had been varied in isolation from one another.^28^ It is important to bear this in mind when interpreting the outcomes.

The thematic groups are shown in Table 7. Each individual parameter is assigned a default value, *C*, a feasible upper extreme, *C^+^*, and a feasible lower extreme, *C^−^*.^29^ Two hundred simulations were conducted with all of the parameters set to the default *C* value and then a further 200 simulations were conducted for each parameter group at its *C^+^* value and then at its *C^−^* value. As there are 11 parameter groups, this yielded 4,600 simulations in all.^30^

**Table 7:** The thematic parameter groups, the parameters they include, their default baseline values (C), and the variation in the parameters relative to the default values.

Parameter group	Parameters included	*C*	*C^−^*	*C^+^*	Percentage change
Animal purchase price	Chicken purchase price	400	320	480	-20 / +20
	Goat purchase price	5000	4000	6000	-20 / +20
	Cattle purchase price	3000	2550	3900	-15 / +30
	Buffalo purchase price	10,000	8500	13,000	-15 / +30
Animal slaughter price	Chicken slaughter price	1200	960	1440	-20 / +20
	Goat slaughter price	7600	6080	9120	-20 / +20
	Female buff slaughter price	28,000	19,600	36,400	-30 / +30
	Male buff slaughter price	42,000	29,400	54,600	-30 / +30
Career advancement probability	Prob. of doing +2 post-school	.5	.42	.57	-15 / +15
	Prob. of obtaining salaried job if completed +2	.25	.17	.32	-15 / +15
	Prob. of advancing to level II from level I salaried job	.75	.64	.86	-15 / +15
	Prob. of advancing to level III from level II salaried job	.5	.43	.58	-15 / +15
	Prob. of advancing to level IV from level III salaried job	.5	.43	.58	-15 / +15
Cash crop price	Potato price per kg	22	18	26	-20 / +20
	Cabbage price per kg	18	14	22	-20 / +20
	Cauliflower price per kg	25	20	30	-20 / +20
Cottage industry income	Daily milk income from cow for one-person household	135	115	155	-15 / +15
	Daily milk income from cow for two-person household	90	77	104	-15 / +15
	Daily milk income from buff for one-person household	225	191	259	-15 / +15
	Daily milk income from buff for two-person household	150	128	173	-15 / +15
	Tomato sale income per harvest	4140	3312	4968	-20 / +20
Day-to-day expenses	Other living expenses	27	22	32	-20 / +20
	Other food expenses	52	42	62	-20 / +20
Educational expenses	Monthly school expenses	400	200	800	-50 / +50
	Monthly +2 expenses	800	400	1600	-50 / +50
Festival, funeral & wedding costs	Festival expenses	200	160	240	-20 / +20
	Wedding gift	100,000	80,000	120,000	-20 / +20
	Funeral cost	200,000	160,000	240,000	-20 / +20
Salaried job abroad	Monthly foreign job salary	10,000	9,000	12,000	-10 / +20
Salaried job in Nepal	Monthly level I job salary	12,000	10,800	13,200	-10 / +10
	Monthly level II job salary	15,000	13,500	16,500	-10 / +10
	Monthly level III job salary	20,000	18,000	22,000	-10 / +10
	Monthly level IV job salary	25,000	22,500	27,500	-10 / +10
Short-term labouring	Daily labouring wage	500	450	600	-10 / +20
	Daily short-term labouring probability	0.19	0.15	0.23	-20 / +20

Eight summary statistics were calculated for each simulation once the runs were completed: (a) the total households in the village at the end of the simulation; (b) the average household size at the end of the simulation; (c) the villager count at the end of the simulation; (d) the combined number of days that households spent in debt over the course of the simulation; (e) the total number of households who were in debt at some point during the simulation; (f) total household cash at the end of the simulation; (g) total household loans at the end of the simulation; (h) the Gini Index for the village at the end of the simulation.^31^ The impact of using each parameter group's *C^+^* and *C^−^* values on these statistics is shown in Table 8 as a percentage change relative to the baseline simulations. The instances where the change was statistically significant are highlighted.

**Table 8:** Change in each statistic relative to the baseline simulations when a parameter sets C^+^ and C^−^ values are used. The bracketed number is the Mann–Whitney U test score. The statistically significant results are shaded. The darker shade shows a positive change, while the lighter shade shows a negative change.

The results of the SA show that the demographic outcomes of the model are by and large insensitive to changes in the parameters that are set out in Table 7. This is not overly surprising as the demographic components of the model are largely (though not completely) isolated from the model mechanisms in which these parameters play a role. The economic processes in the model are, by contrast, much more closely associated with these parameters. Unsurprisingly, therefore, several of the parameter groups do appear to play a significant role in conditioning economic outcomes. The uncertainty/variability in the day-to-day expenses parameters has the greatest impact on the economic outcomes of any of the parameter groups, possibly because these expenses are applicable to all villagers on all of the timesteps. The uncertainty/variability in animal slaughter prices, cash crop prices, and event costs, meanwhile, has roughly half the effect on household cash and household debt days of the day-to-day expenses group. Cottage industry income and short-term labouring are the other two parameter groups which have statistically significant effects of the outcomes considered.

Unsurprisingly, the household loans, debt days, and experience of debt metrics tend to increase and decrease together, with household cash going in the opposite direction. However, the strength of the relationship between the metrics appears to differ from one parameter group to another. This suggests that they affect village economics in somewhat different ways. The shifts that are seen in household Gini coefficient between the parameter groups lends further weight to this idea. Another important observation is that the magnitude of the changes tends to be substantially greater in the case of household loans and the Gini coefficient that in the case of the other metrics. This is the result of certain households becoming trapped in debt spirals whereby the high rate of interest on debts magnifies any initial financial problems they may have had, pulling them deeper and deeper into the red in a non-linear fashion. By contrast, such feedbacks do not play a prominent role in the other metrics.

Given the changes in the statistics are typically of a similar order of magnitude to the parameter variations, the model can be considered unlikely to suffer from highly sensitive non-linearities, and the conclusions drawn from the model can be considered reasonably robust. An important limitation of this SA is that it does not consider interaction effects between parameter groups [29]. Ideally, this will be addressed in future research. However, the SA nevertheless provides an important insight into how the model functions and which parameter groups are particularly influential.

## 6. Using the model

The model code, its associated files, example output, and R scripts for analysing the output are available from University of Leeds at doi.org/10.5518/962 [27]. Before using the model, it is important to note that it differs substantially from standard NetLogo models in its functioning. Firstly, it relies heavily on the R extension to perform some of the more complex operations, such as data synthesis at initialisation. R is therefore required. Secondly, it records an extremely large volume of data to aid subsequent analysis. This recording is done using a bespoke method that calls on the ‘csv’ extension, rather than using the standard methods for outputting data. Thirdly, rather than using NetLogo's BehaviorSpace for managing batch experiments, a bespoke function has been written for this purpose. It can be called using the ‘Conduct a Scenario Sweep’ button. The function itself is named ‘scenario-sweep’. By default, it simulates the eight scenario combinations (see *Sc*) a designated number of times, each time with a different seed (see below). This can be altered in the model code if necessary.

Prior to running the model in its default form, the following needs to be done:

1. The paths to the R scripts must be updated. The paths are set in the ‘to load-r-scripts’ function of the NetLogo code.
2. The path name for the output data needs to be updated. The path is set in the ‘to prepare-data-log’ function. Data logging can be turned on or off using the ‘logData?’ switch on the interface.
3. The paths at the top of the ‘simulateMarriage.R,’ ‘simulateFertility.R,’ and ‘simulateDeath.R’ scripts need to be updated so that they point towards the data in the data folder. These scripts can be found in the ‘r_scripts’ folder.
4. If conducting one run at a time, the scenario chooser menus on the NetLogo interface can be used to set the cash crop and subsistence crop half yield frequency parameters, to set the average fertility parameter, and to determine whether or not to simulate an earthquake. If conducting a scenario sweep, this is not necessary.
5. To change the other parameters from their defaults, update the values that are set in the ‘to initiate-parameters’ function and search for other instances of the variables which may require updating. Note that changes made to these parameters on the interface will be reset when the ‘to initiate-parameters’ function is called on setup, so the changes need to be made directly in the code. Also note that crop yields cannot be set by the user. They are determined by the ‘initiate-future-yields’ function at initialisation.
6. If conducting a scenario sweep, the number of replications that should be run for each scenario combination can be set using the ‘numberOfRuns’ slider on the interface. Each replication uses a different seed.

***To run a single simulation:*** Set the parameter values using the methods explained above, click ‘Setup,’ and then either repeatedly click ‘Step’ to advance one day at a time or click ‘Run Simulation’ to run the model for the period of time determined by the ‘simulationLength’ slider.

***To run a batch of simulations:*** Determine the number of runs to conduct for each scenario combination using the method described above and then click ‘Conduct a Scenario Sweep.’ If a non-standard batch of simulations is desired, adjust the ‘scenario-sweep’ function and any other parameters as required following the previously stated advice.

***Other things to note:***

• A csv file is created for each simulation run if data logging is set to on. By default, the file name shows the earthquake, fertility, and crop variability scenarios that were simulated, as well as the seed. For example, ‘EQ-21-12-01.csv’ stands for the earthquake scenario, the 2.1 fertility rate, the 12/10 crop variability scenario, and seed #1.
• It is advisable to turn off the ‘view updates’ tick box to speed up the simulations.
• To parallelise the simulations, open multiple instances of NetLogo and adjust the ‘scenario-sweep’ function so that different parameter combinations and/or seeds are run in each instance.
• The model is computationally expensive relative to most NetLogo ABMs. It is therefore advisable to run it on relatively powerful devices.
• Each output file is around 30 MB. It is therefore advisable to ensure sufficient storage is available before running large batches of simulations.
• The land holdings of each household can be highlighted by clicking the ‘Highlight a HH’ button after setup and then clicking on the centre of a household in the world window. Click the ‘Highlight a HH’ button again to return to the normal view.

## 7. Adapting the model

The model can potentially be adapted for use in studies besides the one that it was originally designed for. If this is to be done, users should take care to verify that the model behaves as it should after making the intended changes, and they should be aware of the particularities of the fieldsite that was modelled [24]. To aid the verification process, we recommend using the Shiny app that is detailed in *Section 8* as it facilitates ready visual identification of suspect model behaviours.

## 8. Analysing outputs

By default, the model logs a wide range of simulation values to a CSV file, with a separate CSV file being generated for each run. The outputted data includes the scenario details, the annual crop yields, information on significant events that occurred during the simulations, and the status of villager and household agents on each day of the simulation. Example output from the simulations is provided in the ‘example_output’ folder, in the ‘data_analysis’ folder of the supplementary material.

If you have RStudio, the files can be explored using the ‘output_viewer.R’ application. To do this:

1. Open RStudio and install the ‘Shiny’ package if it has not already been installed.
2. Open the ‘output_viewer.R’ script in RStudio and click ‘Run App’.
3. When the app opens, click the ‘Browse…’ button and select a data output file of interest e.g. a file from the ‘example_output’ folder.
4. Once the upload is complete, select one of the tabs to see an overview of the data, bearing in mind that it can take a while to process, even on a relatively high-performance machine. If the seasonal decomposition charts throw an error, try deselecting some households.

The data can alternatively be explored using custom R scripts. Examples are provided in the ‘data_analysis’ folder. By default, the scripts are set up to analyse the 10 example outputs that are included in the supplementary materials. To use the scripts:

1. Open the scripts in RStudio and ensure that the required packages are installed.
2. Update the path names so that they point to the data that is to be analysed.
3. Run the code.

The ‘village_statistics.R’ script calculates key demographic and financial variables for each of the simulations. The ‘financial_trajectory.R’ script plots household finances over time for each scenario combination.

## Supplementary material *and/or* Additional information

The model code, its associated files, example output, and R scripts for analysing the output are available from University of Leeds at doi.org/10.5518/962 [27].

## Declaration of Competing Interest

The authors declare that they have no known competing financial interests or personal relationships that could have appeared to influence the work reported in this paper.

## Footnotes

2 Processes and changes that happen within any given day are “represented by how they make state variables jump from one time-step to the next” ([23], p. 38).

3 The typical short-term labouring wage rate was established during the fieldwork, as was the aggregate village income from short-term labouring during the year proceeding the fieldwork, but substantial variability across the year in terms of short-term labouring opportunities meant that estimating a parameter for the daily short-term labouring probability parameter proved difficult. We therefore ran a series of one-year simulations in order to identify the parameter value which led to the best match between the survey and simulated aggregate short-term labouring income values. This proved to be 19%.

4 The other living expenses parameter is intended to capture expenses that do not fall under the banner of construction, maintenance, education, food, celebrations, funerals, or livelihoods. As with the short-term labouring opportunities parameter, the other living expenses parameter was determined by running a series of one-year simulations in order to identify the parameter value which led to the best match between the survey and simulated aggregate other living expenses values. This proved to be NPR 27.

5 The following days of the year are defined as festival days in the model: 14^th^, 30^th^, 89^th^, 274-280^th^, 282-288^th^, 290^th^, and 303-307^th^.

6 Goats, cattle, and buffalo also consume fodder and left-over food from meals, but this is not explicitly modelled as it does not have cost implications for the households.

7 This is a simplification, in reality the moment that villagers’ transition between career pathways is much more varied.

8 i.e. Sunday to Friday, except on festival days.

9 For simplicity, it is assumed that widows and widowers do not remarry.

10 At the fieldsite, we found that the sum spent on funerals depends on the affluence of the household and the circumstances of the deceased. Expenditure can be as high as NPR 400,000 in the case of some of the wealthier households. For simplicity, however, we assume a standard expenditure of NPR 200,000. Note that households will often need to take out loans to cover this cost.

11 A smoothing constant of 0.25 was used.

12 It is assumed that households try to grow all of their own maize, millet, and wheat as was typically the case in Namsa, rather than relying on markets.

13 It is assumed that households with just one draft animal are able to agree a reciprocal deal with other households with one draft animal to pool their animals as required, thereby avoiding the need to pay for additional animal hire.

14 In Namsa, we saw an increase in the rate of fission following the earthquake as young people who had been living with parents/parents-in-law would often build temporary shelters that were separate from their parent's/parents-in-law's temporary shelter. However, they were not entitled themselves to reconstruction grants so once homes were rebuilt, they tended to move back in with their parents/parents-in-law.

15 This is largely because of the time demands and physical demands that animal rearing entails. Additional factors that shape animal ownership were said to be land sufficiency (i.e. whether the household has enough land to consistently supply the necessary crop fodder from its own farm), the physical strength, knowledge, and preferences of household members, and broader decisions around the household's livelihood strategy. As these factors are less readily quantified within the model, we focus on adult numbers alone.

16 This results in their cash balance being reset to zero.

17 An example of data desensitisation is lowering data item accuracy in a systematic way [1].

18 Note that in contrast to most uses of spatial microsimulation, we start with disaggregated data for the entire population. We draw on the approach for different reasons to those that it is typically employed for.

19 See Müller and Axhausen [17] and Lovelace and Dumont [13] for discussions of the relative merits of various approaches.

21 Viable values are those that are greater than a villager's current age.

22 As with the initialisation of marriage age, viable values are those that are greater than a villager's current age.

24 The three-year difference in half-yield frequency is an arbitrarily chosen figure.

25 This is a simplification as there are known to be distinctions within the categories. However, difficulties obtaining agreed values for each crop during the focus group discussions meant that it is necessary to use the more general group values for which more clear agreement was attained.

26 For additional comparison, the Nepal Living Standards Survey of 2010/2011 [3] placed the nominal average per capita income level in rural areas at NPR 35,923. This includes farm income, non-farm income, remittances, consumption of own-dwelling, own account production income, and other sources. Share of farm income in all household income averaged 28%, while non-farm income constituted 37%, remittance was 17%, and share of own production consumption was 16% ([4], p. 39).

27 This is true even with the survey derived education value corrected for the effect of the mill household's education spending.

28 It is unlikely that the parameter changes within any of the groups will mitigate one another – the groups have been designed to avoid this.

29 These extremes were determined in discussion with the villagers. It should be noted that the extremes do not represent the potential extremes of individual real-world case (e.g. someone getting an exceptionally good price for a chicken), but rather the perceived extremes of the village average cases as determined by uncertainty and variability in those values (e.g. chicken prices during a conceivably very good year).

30 The no-earthquake, low crop variability, 2.1 fertility rate scenario was used for these simulations.

31 This was calculated for households and is a very crude measure of inequality as it does not take into account the different demographic circumstances of each household or non-monetary wealth of the households.

## Footnotes

1 In the unusual case that no such individuals exist within a household, the youngest adult male will be chosen, regardless of whether siblings are still present. If such a person is not available either, the youngest adult female will be chosen, regardless of whether they have been married before.

20 This was the legal age of marriage at the time the model was designed. The legal age of marriage has since been raised to 20. However, the legal marriage age is not strictly observed in Nepal so this change in law is not necessarily all that problematic.

23 Human mortality is assumed to be zero in the model. While one person died in a neighbouring village, none of the Namsa villagers died, and the earthquake mortality rate for Dolakha district was just 0.09% [19].
